# Animal studies for the evaluation of in situ tissue-engineered vascular grafts — a systematic review, evidence map, and meta-analysis

**DOI:** 10.1038/s41536-022-00211-0

**Published:** 2022-02-23

**Authors:** Suzanne E. Koch, Bente J. de Kort, Noud Holshuijsen, Hannah F. M. Brouwer, Dewy C. van der Valk, Patricia Y. W. Dankers, Judith A. K. R. van Luijk, Carlijn R. Hooijmans, Rob B. M. de Vries, Carlijn V. C. Bouten, Anthal I. P. M. Smits

**Affiliations:** 1 Department of Biomedical Engineering, Eindhoven University of Technology, Eindhoven, The Netherlands; 2 Institute for Complex Molecular Systems (ICMS), Eindhoven University of Technology, Eindhoven, The Netherlands; 3 SYstematic Review Centre for Laboratory animal Experimentation (SYRCLE), Department for Health Evidence, Radboud Institute for Health Sciences, Radboud UMC, Nijmegen, The Netherlands

**Keywords:** Tissue engineering, Regenerative medicine, Implants, Preclinical research, Vascular diseases

## Abstract

Vascular in situ tissue engineering (TE) is an approach that uses bioresorbable grafts to induce endogenous regeneration of damaged blood vessels. The evaluation of newly developed in situ TE vascular grafts heavily relies on animal experiments. However, no standard for in vivo models or study design has been defined, hampering inter-study comparisons and translational efficiency. To provide input for formulating such standard, the goal of this study was to map all animal experiments for vascular in situ TE using off-the-shelf available, resorbable synthetic vascular grafts. A literature search (PubMed, Embase) yielded 15,896 studies, of which 182 studies met the inclusion criteria (*n* = 5,101 animals). The reports displayed a wide variety of study designs, animal models, and biomaterials. Meta-analysis on graft patency with subgroup analysis for species, age, sex, implantation site, and follow-up time demonstrated model-specific variations. This study identifies possibilities for improved design and reporting of animal experiments to increase translational value.

## Introduction

Cardiovascular disease (CVD) is one of the leading causes of patient morbidity and mortality, accounting for approximately one third of all deaths globally^1,2^. Treatment of cardiovascular disorders that require bypassing of blood vessels due to occlusion or narrowing places a large clinical demand on vascular substitutes^3^. The gold standard for vascular substitutes is the autologous blood vessel. However, dependent on the pathophysiological state of the patient, using autologous vessels is not always possible^4^. Alternatively, patients often receive non-resorbable synthetic vascular grafts made from expanded polytetrafluorethylene (ePTFE, i.e., GoreTex) or polyethylene terephthalate (PET, i.e., Dacron), which have been clinically approved for large diameter blood vessels since 1956^5^. Although these non-resorbable synthetic replacements demonstrate good patency rates and long-term performance for replacement of aorta and larger arteries, they perform poorly in small diameter (<6 mm) blood vessel applications, with high rates of infection and thrombosis^6,7^. With the aging population, and thus the expected rise in CVD incidence, there is a growing clinical demand for improved vascular replacement grafts^1^.

Over the last decades, tremendous progress has been made in the development of bioresorbable tissue engineered vascular grafts (TEVG) as an alternative to non-resorbable synthetic grafts^7–9^. Upon implantation, these grafts are aimed to temporarily take over blood vessel functionality and induce functional endogenous tissue regeneration, while they are gradually resorbed, directly in the tissue’s functional site, or in situ^7,10^. This approach, also known as in situ vascular tissue engineering (TE) offers translational benefits in terms of costs and logistical complexity when compared to traditional in vitro TE strategies, as grafts for in situ TE are off-the-shelf available and circumvent lengthy in vitro culture protocols^11^.

Preclinical testing of newly developed in situ TEVGs in animal models is commonplace^12^, placing a heavy burden on laboratory animals. Albeit that the influence of animal species and age on vascular graft acceptance has been described decades ago^13^, no consensus or standard has been defined for animal models to be used, nor for read-out parameters to be measured. As the approach of in situ TE is intrinsically dependent on the host response to the implanted graft, TEVG outcome may depend on model-specific factors that influence this host response. For example, recent research emphasizes that animal sex and implantation site have a significant effect on TEVG outcome^14–16^. This makes any comparison between animal studies difficult and, combined with the species-specific differences of clinically relevant processes (e.g., trans-anastomotic endothelialization)^17^, may explain why clinical translation has been inefficient. Therefore, the overall goal of this systematic review was to map all animal studies evaluating in situ TEVGs based on resorbable synthetic grafts that have been reported to date. With that, we aimed to identify potential model-specific variations in the evaluation of in situ TEVGs, in order to improve the quality (and reporting) of animal experimentation for in situ TEVGs.

We systematically collected data from all in vivo studies published on in situ TEVGs, including both small and large animals. In situ TEVGs were defined as primarily synthetic, fully degradable, and off-the shelf available grafts, which were implanted as vascular interposition graft. A meta-analysis was performed for patency, representing the functionally most important outcome measure, and the effects of species, sex, implantation site, animal age, and follow-up time on patency were assessed via subgroup analyses.

## Results

### Literature search and screening

**Fig. 1:**
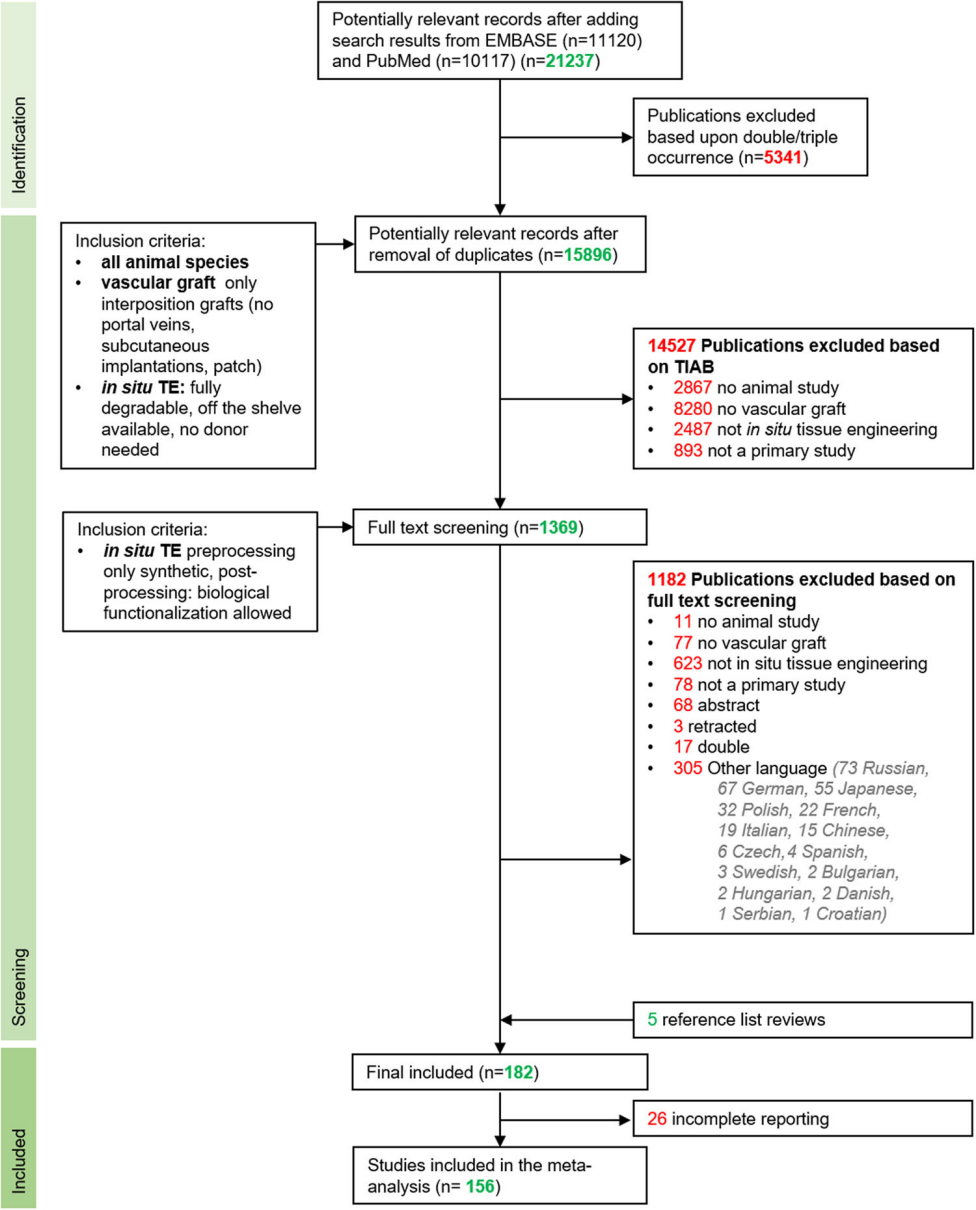
PRISM flow chart of study selection process. The systematic search in Pubmed and EMBASE yielded 15,896 unique publications. After title and abstract screening, articles were screened full text of which 1,182 articles were excluded based on the exclusion criteria. Data from 182 articles (see Supplementary Reference List) was extracted and because of incomplete reporting (e.g., animal number not reported) in 26 articles, 156 articles were included in meta-analysis. Abbreviations: TE tissue engineering.

The bibliographic search strategy (Supplementary Tables 1 and 2) retrieved 21,237 articles in total, including 10,117 from PubMed and 11,120 from Embase (Fig. 1). After the removal of duplicates, 15,896 unique articles were screened on titles and abstracts. During this title and abstract screening, 14,527 studies were excluded, leaving 1,369 articles for full-text screening. After full-text screening, 182 articles were included in the review (Supplementary Reference List), of which 156 articles were eligible for meta-analysis.

### Mapping of animal models and study characteristics

**Fig. 2:**
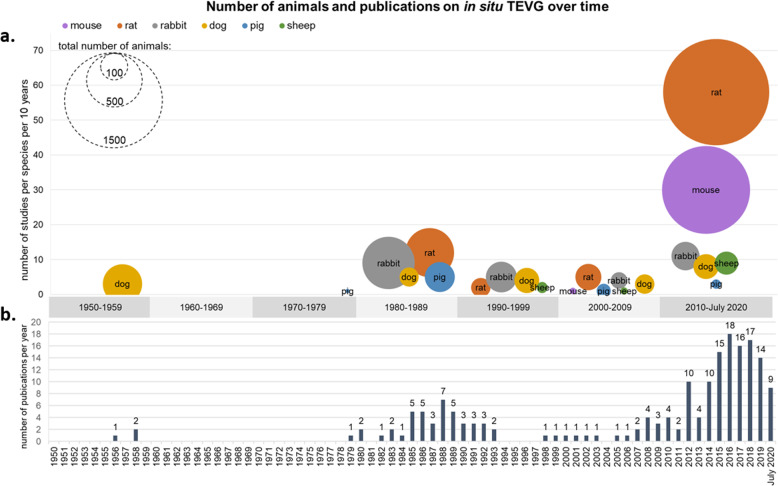
Number of animals and publications on in situ TEVG. **a** Number of studies and number of animals per species combined per decade. Size of the dot represents total number of animal used (total number of animals per publication, e.g., in situ TEVG and control grafts), location of the dot on *y*-axis represents total number of studies published per decade per species. **b** Number of publications on animal studies of in situ TEVG per publication year. Abbreviations: TEVG Tissue Engineered Vascular Graft.

The 182 included studies reported a wide variety of study designs, animal characteristics, and materials (Supplementary Data 1). Overall, a total number of 5,101 animals were used. The first studies including bioresorbable vascular interposition grafts were published between 1956-1958 by Harrison et al. (Fig. 2)^18–20^. In those studies, (resorbable) vascular grafts were assessed in a dog model. Graft resorption was, however, reported as an unintentional finding, rather than a preconceived design feature. During the 1980s, two main research groups, Greisler et al. and Van der Lei et al., studied synthetic vascular grafts that were designed to degrade in situ, mainly using rabbit and rat models. After a period of relatively few publications, there was a surge in reports on degradable vascular grafts around the year 2000. From this moment on also mice were used as implantation model. Over the last decade, a steep rise in publications on in situ TEVGs can be observed, in which rats and mice are predominantly used as the animal model, both in terms of number of animals and in number of publications.

**Fig. 3:**
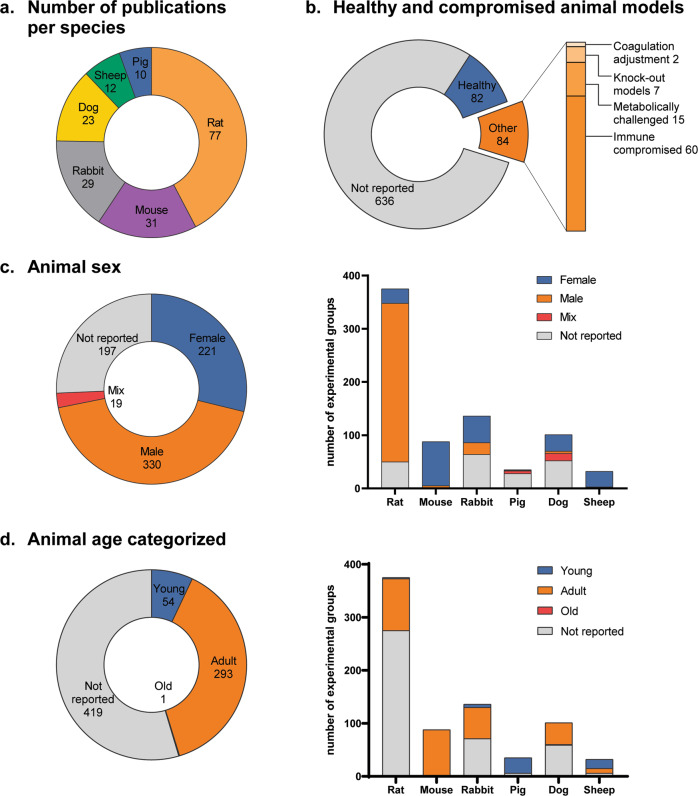
Mapping of study characteristics included in the systematic review. **a** Number of publications per species of all included studies. **b** Number of subgroups reporting health status of animals all included studies. Compromised animal models include; coagulation adjustments (e.g., platelet inhibitor treatment), Knock out (KO) models (e.g., Myeloid specific PDGF KO, CCR2 KO), metabolically challenged models (e.g., Diabetic animals), and immune compromised animals (e.g., SCID/beige, athymic). **c** Reported animal sex, and **d** animal age of all experimental groups and categorized per animal species according to cut-off values described in Table 1.

Overall, small-animal models were used in most study designs, with 42% of all publications using rats and 17% using mice (Fig. 3a). Other animal models used were rabbits (16%), dogs (13%), sheep (7%), and pigs (5%). In absolute numbers of animals, however, sheep were used the least as animal model for in situ TEVG (Supplementary Data 1). For 3,201 animals, health or immune status was not reported (Fig. 3b, 636 experimental groups). However, 459 animals (82 experimental groups) were specifically reported as being healthy and 562 animals (84 experimental groups) were reported as diseased, most of which were immune-compromised (60 experimental groups), such as severe combined immunodeficiency (SCID)/beige mice. Six articles studied metabolically challenged animals, including diabetic and hyperlipidemic animals.

For all studies combined, female and male sexes seem evenly distributed (Fig. 3c). However, a species-dependent bias was present within this data; rats were predominantly male, whereas mice and sheep were most often female. Animal sex was not reported for 197 experimental groups from 54 studies, involving 1,338 animals (26% of all included animals), of which most were rabbit, pig, and dog models.

In light of variation in life expectancy, species-dependent categorization of animal age and follow-up time was performed based on average life expectancy and age of puberty, respectively (Table 1). Animal age was categorized as ‘young’ (<puberty) or ‘adult’ (≥puberty) per animal species^21–25^. Of the publications reporting animal age, most of the animals used were of adult age at the moment of TEVG implantation (293 experimental groups, 38%) and only few animals were young (54 experimental groups, 7%) (Fig. 3d). One study specifically reported on the use of old animals, testing TEVGs in elderly beagles, which was defined as older than 3 years^26^. Age was not reported in the majority of the studies (419 experimental groups, 55%), and animal age was least reported for rats.

**Table 1:** Species-dependent subcategorization of age and follow-up time for data mapping and meta-analysis.

	Age puberty (average species)	Cut-off value young vs adult	Total life expectancy (average species)	Cut-off value short vs medium follow up (1.67% of total life expectancy)	Cut-off value medium vs long follow up (6.67% of total life expectancy)	References
Mouse	5–7w	5w	2y	12d	1.6m	^21,22^
Rat	7–11w	7w	3y	18d	2.3m	^22,23^
Rabbit	3–6m	3m	10y	60d	7.9m	^22,24,25^
Pig	4–6m	4m	15y	3m	1y	^22^^,88^
Dog	5–7m	5m	14y	85d	11.8m	^22^^,88^
Sheep	n.a.^a^		12.5y	76d	9.9m	^22,88^

^a^For sheep studies: method description mentioning “juvenile” or “lamb” was sufficient to make the categorization

**Fig. 4:**
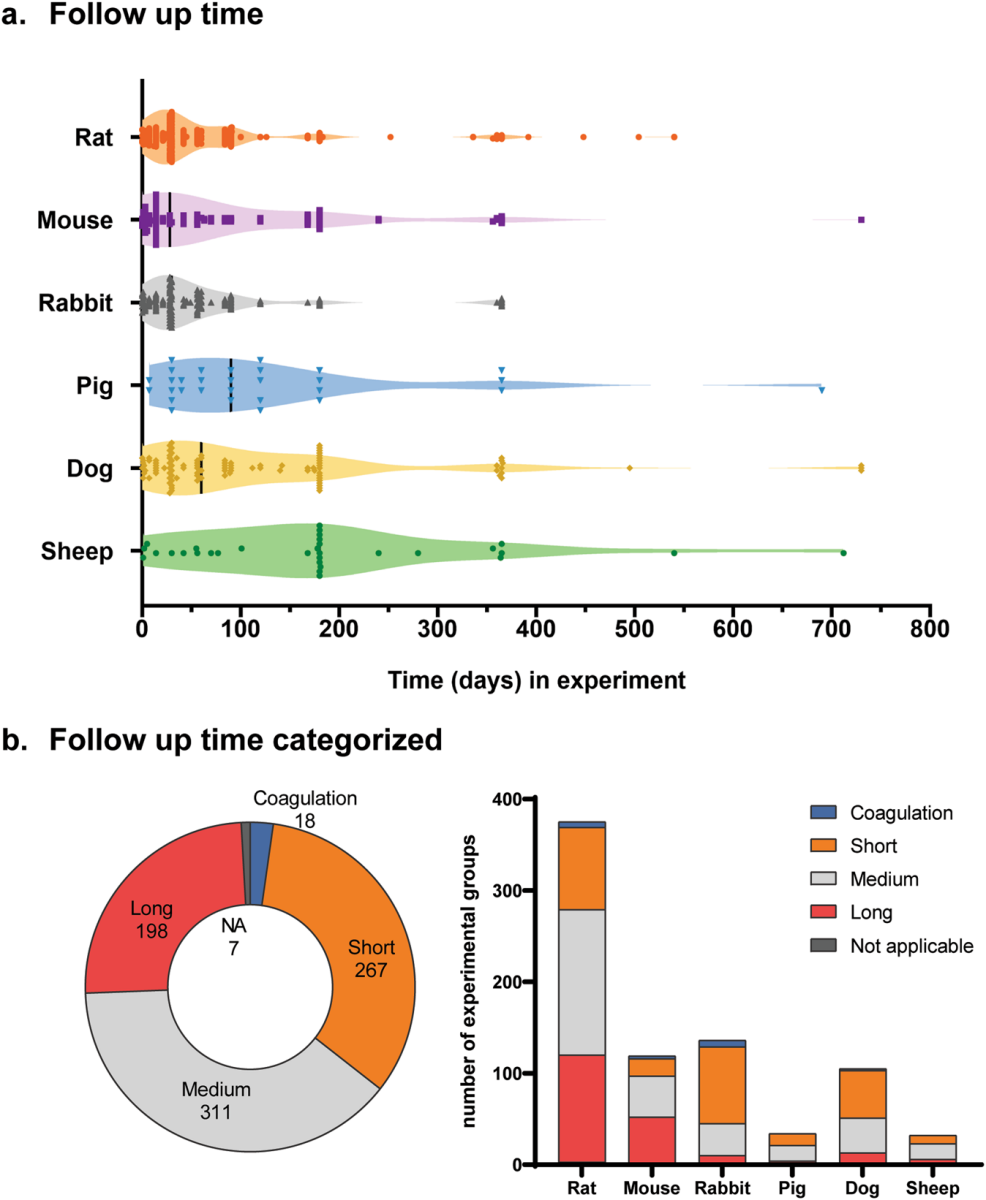
Mapping of study characteristics included in the systematic review. **a** Follow-up time in days for all experimental groups categorized per species, black vertical line indicates average. **b** Follow-up time of all experimental groups and categorized per animal species according to cut-off values described in Table 1.

Mapping of follow-up time showed a higher average follow-up time for larger animal models; 188 days in sheep, 131 days in pigs, and 124 days in dogs compared to 70 days in rats and 85 days in mice (Fig. 4a). Interestingly, in view of total lifespan (Table 1), average follow-up time in rabbits was relatively short (63 days). For all species, experiments with a follow up time of 1 year were performed; however, only 12 publications exceeded this implantation time.

Categorizing follow-up time according to species average life expectancy (Table 1) resulted in an even distribution of experimental groups between short (267), medium (311), and long (198) follow-up times (Fig. 4b). Few studies included follow-up times shorter than 1 day, which mainly focused on coagulation rather than on in situ regeneration. For only 7 experimental groups, follow-up time was not clearly described.

**Fig. 5:**
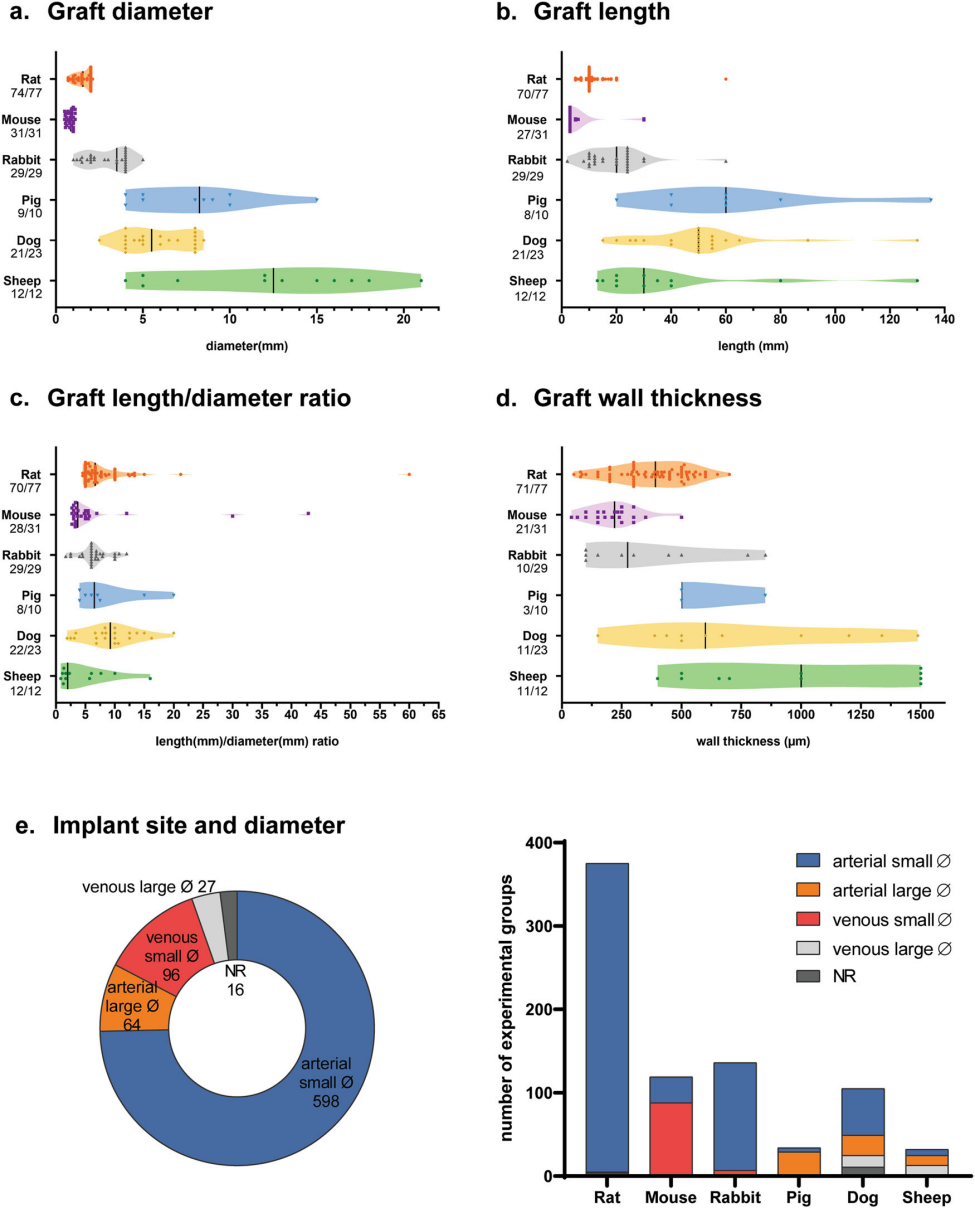
Mapping of study characteristics included in the systematic review. **a** Graft diameter (in mm) per study per species. Numbers below species indicate n publications describing this characteristic/total publications. **b** Graft length (in mm) per species. **c** Graft length/diameter ratio per species. **d** Graft wall thickness (in µm) per species. **e** Implant site (arterial, venous) and small (<6 µm) and large (>6 µm) inner diameter. Abbreviations: NR Not Reported.

In addition to animal and study design aspects, graft characteristics were mapped per study per species (Fig. 5). Generally, average graft diameter was larger for grafts implanted in pigs (7.9 ± 3.5 mm), dogs (5.9 ± 1.8 mm) and sheep (12 ± 5.7 mm) when compared to rabbits (3.0 ± 1.2 mm), rats (1.6 ± 0.44 mm), and mice (0.80 ± 0.20 mm). However, in all species small-diameter vascular grafts (diameter < 6 mm) have been implanted (Fig. 5a). Similarly, graft length was on average longer for grafts implanted in pig (62 ± 35 mm), dog (51 ± 24 mm) and sheep (39 ± 34 mm) models when compared to rabbit (19 ± 11 mm), rat (12 ± 6.8 mm), and mouse (5.3 ± 7.2 mm) models (Fig. 5b). The ratio between graft length and diameter was above the previously described threshold of 10 for false positive patency in few studies only (Fig. 5c)^27–29^. Graft wall thickness was variable between studies, with the highest average for sheep (978 ± 454 µm) and dogs (752 ± 436 µm). However, of note, less than half of the studies reported wall thickness for all species except for rats and mice, (Fig. 5d). To compare graft flow conditions, all experimental groups were subcategorized based on arterial and venous implant site as well as small (<6 mm) and large (>6 mm) graft diameter. Implantation of arterial small-diameter grafts (76% (598/792 experimental groups)) was most common, followed by venous small-diameter grafts (12% (96/792)), arterial large-diameter grafts (8%, (64/792 experimental groups)), and venous large-diameter grafts (3% (27/792 experimental groups) (Fig. 5e). Notably, in rats most TEVGs were classified as arterial small-diameter grafts (99% (370/375 experimental groups), whereas in mice most implants were venous small-diameter grafts (74% (88/119 experimental groups).

**Fig. 6:**
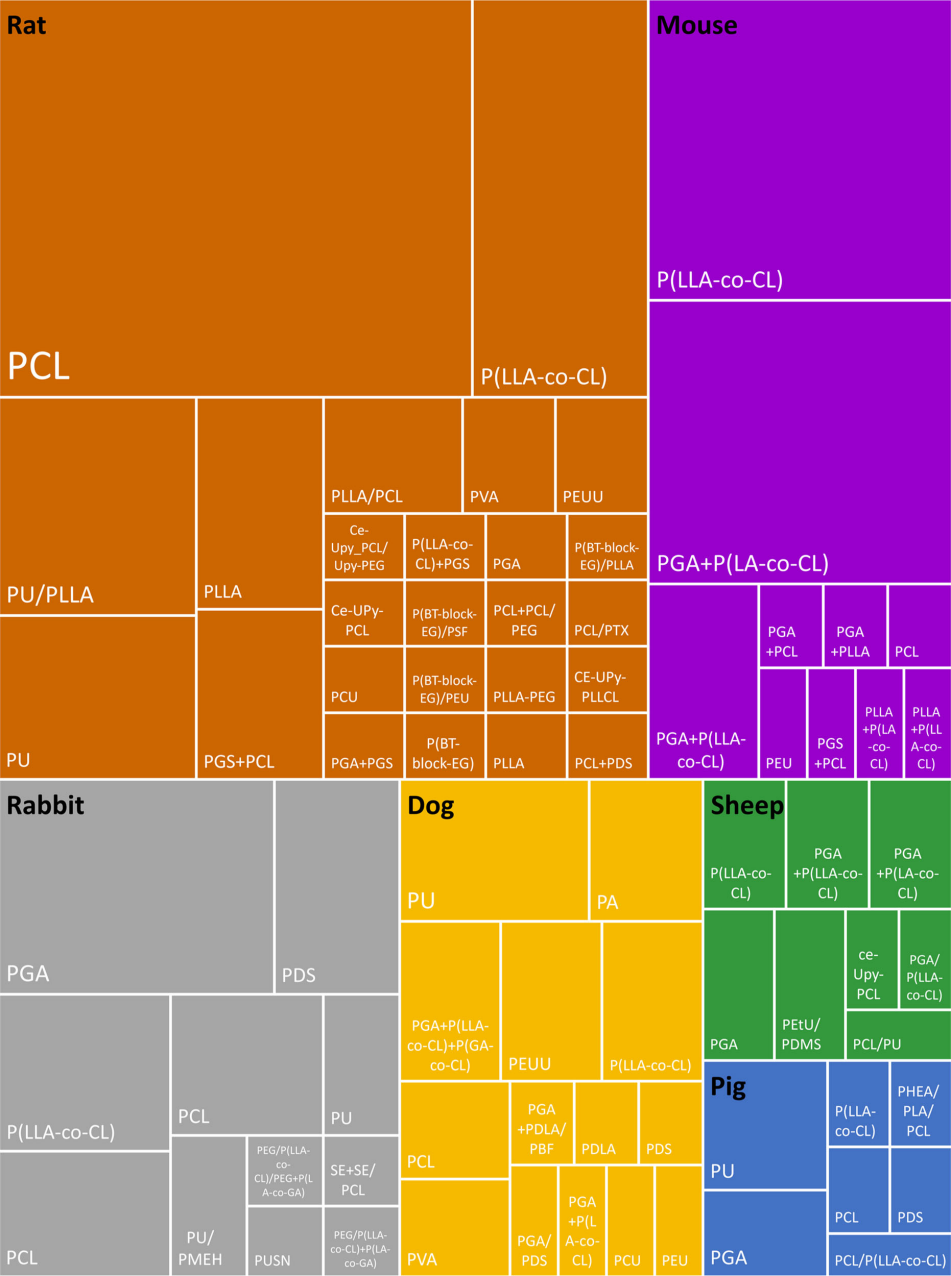
Mapping of materials used for in situ TE vascular grafts per species. Relative size represents number of studies using the specific material type. Blended materials are separated by /, and different graft layers are separated by +. Abbreviations: PHEA a,b-poly(N-2-hydroxyethyl)-D,L-aspartamide, CE-Upy Chain extended-ureido-pyrimidinone, PTX Paclitaxel, PBF poly(2,3-butylene furmarate), PMEH poly(2-methacryloyloxyethyl phosphorylcholine-co-2-Ethylhexyl methacrylate), PUSN poly(ester urethane)urea with disulfide and amino group, PetU poly(ether)urethane, P(LA-co-GA) poly(lactid acid-co-glycolic acid), P(LLA-co-CL) poly(L-Lactic-co-caprolactone), P(BT-block-EG) poly(polybutylene terephthalate-co-polyether glycol), PA polyamide/nylon, PCL polycaprolactone, PCU polycarbonate urethane, PDMS polydimethylsiloxane, PDS polydioxanone, PDLA poly-D-L-lactic acid, PEU polyesterurethane, PEUU polyesterurethane urea, PEG polyethylene glycol, PGA polyglycolic acid, PGS polyglycerol sebacate, PLLA poly-l-lactic acid, PU polyurethane, PVA polyvinyl alcohol, SE synthetic elastin.

Finally, the materials used to produce TEVGs were mapped. Within all included studies, 48 unique combinations of materials (see Supplementary Table 3 for examples and commercial names), composites and blends were studied, ignoring differences in polymer ratios or manufacturing details (Fig. 6). In addition, 271 experimental groups included testing of a form of biofunctionalization, with 46 unique functionalization types in total, of which fibrin and heparin were applied most. Categorization by species showed that polycaprolactone (PCL) was most often tested material in rat models, whereas poly(l-lactic acid-co-caprolactone (P(LLA-co-CL)) was most often tested material in mouse, sheep, pig, and dog models. In rabbits, polyglycolic acid (PGA) was tested most often.

**Fig. 7:**
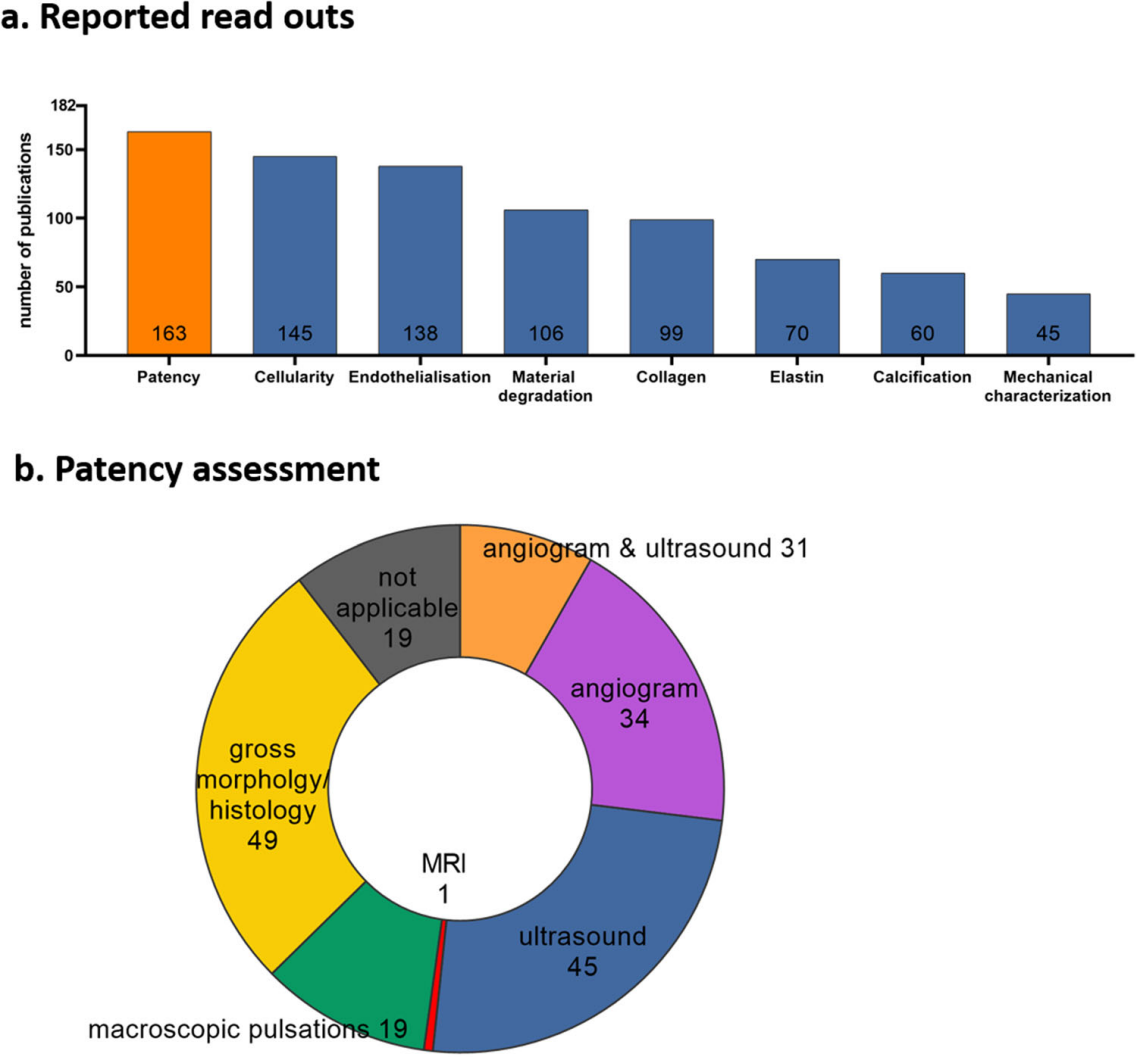
Mapping of reported read-outs for preclinical TEVG assessment and patency assessment methods. **a** Number of studies reporting on read-outs; patency, cellularity, endothelialization, material degradation, collagen formation, elastin formation, calcification, and mechanical characterization. **b** Number of studies per patency assessment method; combination of both angiogram and ultrasound, angiogram, ultrasound, magnetic resonance imaging (MRI), macroscopic observation for pulsations distal to graft upon explantation, gross morphology upon explantation and histology, or not applicable (number of studies not reporting on patency).

With respect to readout parameters, patency (163 publications), cellularity (145) and endothelialization (138) were reported in most studies, whereas material degradation (106) and collagen deposition (99) were described in about half of the publications (Fig. 7a). Less studies reported on elastin formation (70), calcification (60) and mechanical properties of the explanted graft (45). Additionally, some studies reported other research-question-specific read outs (e.g., thrombotic potential of the material, mechanical testing of the material, assessment of the origin of infiltrating cells). Mapping of patency assessment methods showed that 31 of 182 studies assessed patency via a combination of both angiography and ultrasonography, 34 studies applied angiography, and 45 studies used ultrasound only (Fig. 7b). However, most studies assessed patency via gross morphology inspection combined with histology (49 studies).

### Quality assessment

**Fig. 8:**
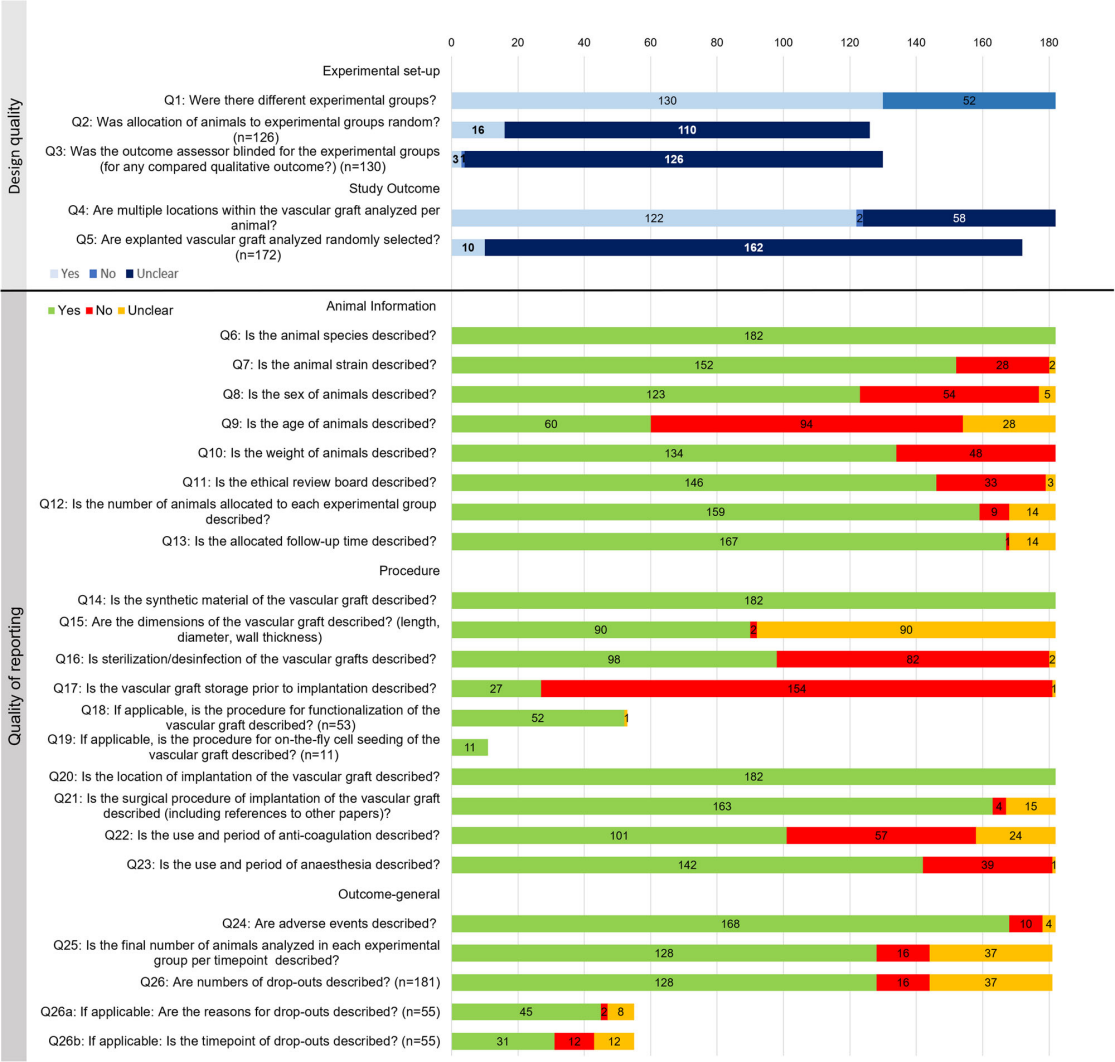
Assessment of quality of study design and quality of reporting. For all 182 included studies quality was assessed with 26 questions related to experimental set-up, study outcome design, animal information, procedure, and general outcome (see Supplementary Table 4 for the definition of separate answers per study).

Key points for assessment of design and reporting quality were assessed for all 182 included studies (Fig. 8 and Supplementary Data 2). Although 130 studies included multiple experimental groups (Q1), only 16 studies reported random allocation of animals to these experimental groups (Q2). Four studies were designed to implant both in situ TE graft and control graft within the same animal, for which defining random allocation was not applicable. Only 3 studies reported blinding of the outcome assessor for qualitative analysis (Q3) and only 10 studies reported randomized analysis of explanted grafts (Q5). Within 122 studies, however, analysis of multiple locations within the vascular grafts was reported (Q4).

All studies reported animal species (Q6) and 84% of studies reported the animal strain (Q7). However, other basic animal characteristics such as animal sex (70%) (Q8), animal weight (74%) (Q10), and especially animal age (33%) (Q9) were reported less frequently. The number of animals allocated to each experimental group (87%) (Q12), follow-up time per experimental group (92%) (Q13), and ethical considerations (80%) (Q11) were relatively well reported. Synthetic graft material (Q14) and implantation site (Q20) were reported in all included studies. However, only 50% of the studies reported main graft dimensions, i.e., graft length, diameter, and wall thickness and two reported none of these parameters (Q15). The graft sterilization method (Q16) was reported for 45% of the studies and the storage procedure prior to implantation (Q17) was only reported in 15% of the studies. When biofunctionalization (Q18) or on-the-fly cell pre-seeding (Q19) was applied, the methods were often well-described (98% (52/53) and 100% (11/11) studies, respectively). Additionally, the surgical procedure (Q21) was relatively well described or referred to previous articles (90%) and more than half of the papers also described the use of anti-coagulation (55%) (Q22) and anesthesia (78%) (Q23). Adverse events were described in most studies (92%) (Q24). The final number of animals per experimental group per timepoint (Q25) was reported in 71% of the studies. For the studies describing drop-outs (Q26), the reason for animal drop-out (82% (45/55)) (Q26a) was described more often than the timepoint of drop-out (56% (31/55)) (Q26b).

### Meta-analysis

**Fig. 9:**
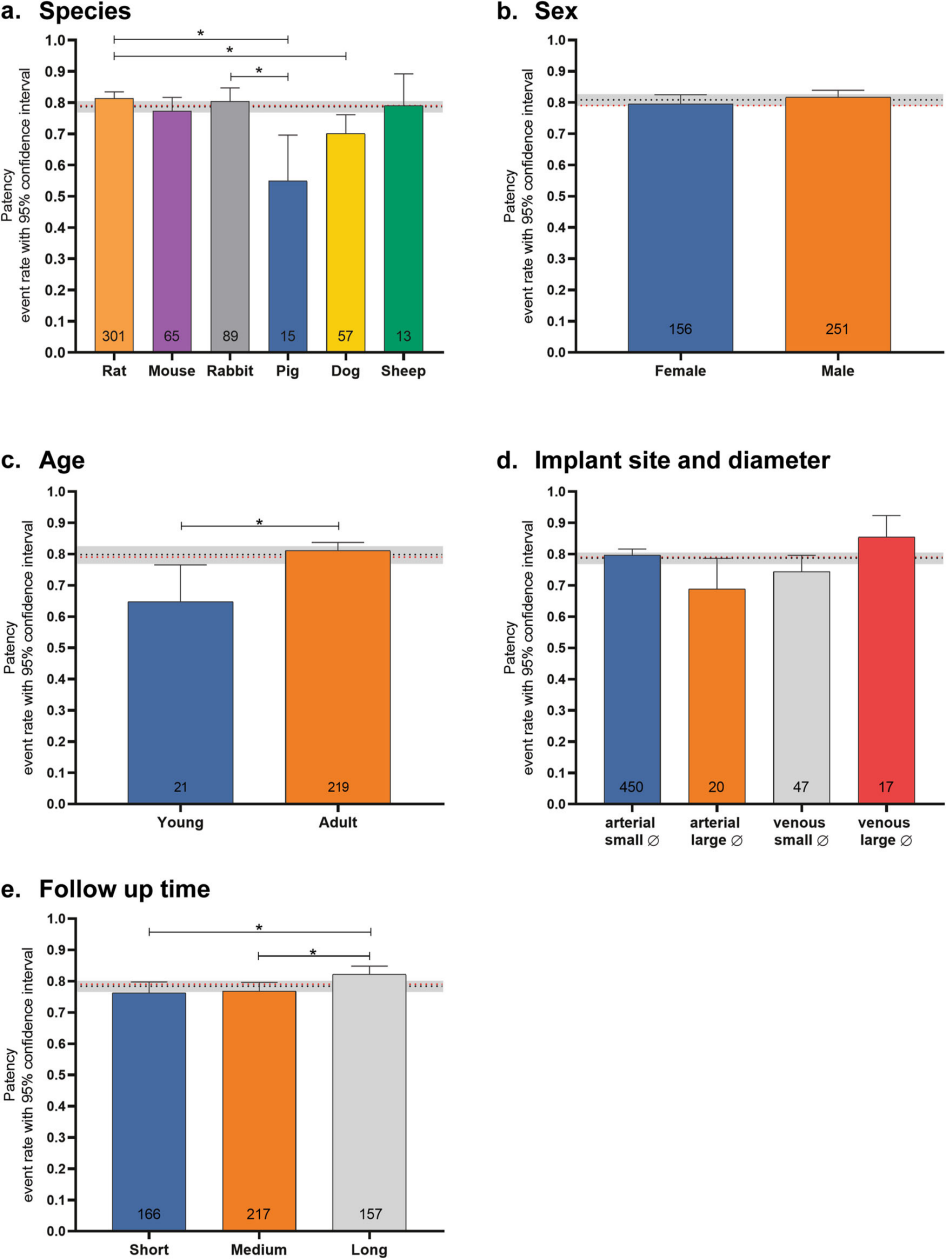
Meta-Analysis and subgroup analyses. **a** Subgroup analysis for species, **b** sex, **c** age, **d** implant site and diameter, and **e** follow-up time. Age and follow-time subcategorization are species-dependent; see Table 1 for cut-off values *young vs adult* (**c**) and *short vs medium vs long* (**e**) per species. Numbers in bar represent number of experimental groups. Black dotted line and gray shades representing grouped ER with 95% CI respectively. Red dotted line: overall ER patency. Significance *p < 0.05.

A meta-analysis was performed on graft patency, representing the primary functional outcome parameter of vessels and vascular implants. For the 156 studies (earliest study 1958, latest study July 2020) that were eligible for meta-analysis (e.g., reported both patency rate and number of animals per experimental group), including a total of 540 experimental groups and *n* = 3,389 animals, an overall patency rate of 0.79 (95%CI [0.77–0.8]; I^2^ 19%) was found (Supplementary Fig. 1). To investigate if animal study characteristics influenced study outcome, subgroup analyses were performed to assess the effect of species, sex, implantation site (pre-specified subgroups), animal age, and follow-up time (post hoc defined subgroups) on patency (Fig. 9). Due to the large variation in biomaterials used (Fig. 6), no subgroup analysis on material type could be performed because of a too low number of experimental groups per material type. Subgroup analysis demonstrated a significantly lower patency rate of 0.54 in pigs ([0.39–0.70; I^2^ 61%) compared to 0.81 in rats ([0.79–0.83]; I^2^ 0%) and 0.83 in rabbits ([0.75–0.85]; I^2^ 5.7%). Additionally, the patency rate in dogs (0.70 [0.63–0.76]; I^2^ 12%) was significantly lower when compared to rats. No significant differences in patency rate were found for mice (0.77 [0.72–0.82]; I^2^ 57%) and sheep (0.79 [0.63–0.89]; I^2^ 25%) (Fig. 9a). Patency rate did not significantly differ between male and female animals (Fig. 9b). Subgroup analysis on the influence of relative animal age indicated a significantly higher patency rate in adult animals (0.81 [0.78–0.84]; I^2^ 31%) when compared to young animals (0.65 [0.51–0.77]; I^2^ 36%) (Fig. 9c). Analyzing the effect of implantation site combined with graft diameter (cut-off 6 mm) demonstrated no significant differences in patency rate between arterial large-diameter grafts (0.69 [0.57–0.79]; I^2^ 14%), arterial small-diameter grafts (0.79 [0.77–0.82]; I^2^ 3%), venous large-diameter grafts (0.85 [0.74–0.92]; I^2^ 0%), and venous small-diameter grafts (0.74 [0.68–0.80]; I^2^ 67%) (Fig. 9d).

Lastly, the influence of relative follow-up time, normalized to the average animal life expectancy per species, was analyzed, which demonstrated a significantly higher patency rate within experimental groups with a long follow-up time (0.82 [0.79; 0.85]; I^2^ 1.2%) compared to short (0.76 [0.73–0.80]; I^2^ 3.8%) and medium (0.77 [0.74–0.80]; I^2^ 24%) follow-up times (Fig. 9e).

Sensitivity analyses for merging of experimental groups containing 1 animal and subcategorization based on species, age and follow-up time (Supplementary Fig. 2) showed similar trends in patency rates for all analyses. For species-dependent sensitivity analysis of animal age no change in significance occurred with the exclusion of a species (Supplementary Fig. 3). Only for species-dependent implant site and diameter, the exclusion of mice changed the trends towards a significantly higher patency rate for venous small-diameter implants (0.96 [0.90–0.99]; I^2^ 65%) compared the other groups. However, it should be noted that with exclusion of mice, the venous small-diameter group contained only 2 experimental groups. (Supplementary Fig. 4). Similarly, the exclusion of large diameter vascular graft from subgroup analysis on species enhanced significant differences between species, with only 4 and 5 experimental groups for sheep and dog, respectively (Supplementary Fig. 5). For both sensitivity analyses, these limited experimental group numbers prevented any robust statistical analysis.

## Discussion

The overall goal of this systematic review and meta-analysis was to map and analyze all animal experiments that have been reported to date for in situ TEVG based on resorbable synthetic scaffolds. Moreover, we aimed to identify model-specific parameters that could potentially influence outcome and define points-of-attention in the design of animal experiments for in situ TEVGs and the reporting thereof. Meta-analysis was performed on patency as a clinically relevant and main functional readout parameter for TEVGs. The main findings are that for the preclinical evaluation of in situ TEVGs rats are the most often used, choice of animal sex and implant site are often dependent on animal species, and both health status and animal age are poorly reported. Meta-analysis with subsequent subgroup analysis showed that patency rates for TEVGs are influenced by follow-up time, as well as the choice of animal species and animal age.

### Number of publications and species

The use of resorbable synthetic grafts to induce tissue regeneration in situ has gained tremendous momentum over the last decade for a broad variety of applications^10,30^. This is driven by the recognition that the inflammatory response to an implanted material can be modulated to induce and steer the formation and remodeling of functional new tissue^10,16^. This trend has placed more emphasis on animal experimentation in order to test the in vivo response to newly developed materials already at an early stage. This is reflected by the mapping of in vivo studies on resorbable synthetic vascular grafts as performed in this study, showing a strong increase in the number of publications on in situ TEVGs in the last decade.

Moreover, our analysis shows a clear shift towards rat and mouse models as the most frequently used animal models to test TEVGs for in situ TE. Particularly, a steep rise in the use of mice over the recent years can be observed. This is probably due to the development of knockout and transgenic techniques, which allow assessment of TEVG functionality in simulated pathologies already early in preclinical stage, or assessment of TEVGs seeded with non-autologous cells^31,32^. The largest part of the immune-compromised animals included in this systematic review were SCID/beige mice (73% of all immune-compromised animals), which are often used to circumvent the adaptive immune response when testing TEVGs seeded with non-autologous cells such as human cells^32^. In this systematic review, we only included animals of experimental groups that used TEVGs without cells (this was usually the ‘control group’ in the studies testing TEVGs seeded with human cells), or with on-the-fly seeded cells (i.e., no in vitro cultures), which suggests that the number of immunocompromised mice used for TEVG research in general is even larger.

### Interspecies differences

Given that in situ vascular TE is heavily dependent on the intrinsic regenerative capacity of the recipient of the TEVG, the choice of animal model may be of crucial importance for the translational relevance of the outcome. While much emphasis has been placed on the influence of local graft properties (e.g., material choice, mechanical properties, microstructure)^7,33^, the potential systemic influences that arise from the choice of in vivo model have been largely underrepresented in the literature. Both inter-species differences in the regenerative capacity and processes, as well as intra-species characteristics such as age, sex, and multifactorial disease profiles, are important considerations for the in vivo testing of in situ TEVGs^16^. The meta-analysis performed in this study revealed an overall median patency of 79%, which is in the same range as the median patency reported in a recent systematic review of Skovrind et al., who observed an overall patency of 83% for TEVGs (seeded/unseeded, biological/hybrid/synthetic and degradable/non-degradable) in large-animal models only^34^. Albeit that we cannot conclude which animal model is most representative for the clinical situation, we detected inter-species differences in overall patency rates. Interestingly, significantly lower patency rates were found in dogs and pigs when compared to rats, and significantly lower patency rates in pigs were observed when compared to rabbits. The main compromising factors for TEVG patency are thrombus formation and adverse remodeling (i.e., intimal hyperplasia). In this systematic review, we did not quantify to what extent the reasons for graft occlusion were due to either cause and whether this was subject to inter-species differences. As the reporting on the reason and timepoint of graft failure was incomplete in part of the papers, it would be too speculative to make the direct link. Nevertheless, important inter-species differences have previously been reported with respect to thrombogenicity, as well as regenerative potential^35–37^.

With respect to inter-species differences in thrombogenic potential, Grabowski et al. (1977), assessed platelet adhesion of heparinized blood of eight different species on different biomaterials under flow. It was observed that the inter-species differences were biomaterial dependent, meaning there was a much higher platelet adhesion of dog blood compared to human (and cow, baboon, macaque, pig, and sheep) to “Cupropjan” and “Avcothane” biomaterials, but this dog-human difference was absent for GoreTex biomaterials^35^. Similarly, in one of the earlier papers included in our analysis, Van Der Lei et al. (1989) describe species-dependent thrombogenicity of PU grafts^36^. Specifically, implantation of PU vascular grafts in the rabbit carotid artery was compared with their previous studies with the same prostheses in the rat aorta^38^. Their results show that prostheses that remained patent in the rat model, occluded in the rabbit model. Albeit that there were two different sites used, the latter more closely resembled the clinical situation in terms of thrombogenicity according to the authors^36^, as well as a review by Byrom et al.^39^. Almost 30 years later, differences in hemostasis and thrombosis between preclinical animal models and humans are still extensively studied, because extrapolating the preclinical findings to humans still results in inaccurate predictions of the clinical scenario^37^.

Another important contributor to the inter-species differences in patency may be differences in regenerative capacity between animals. For example, Zilla et al. previously reported on the importance of differences in endothelialization potential between animal models. Particularly, transanastomotic endothelialization is dependent on species, senescence, anatomical dimensions, and graft surface, with species being the most important determinant^17^. Another important determinant of success for in situ TEVG is the balance between tissue regeneration and resorption of the initial synthetic implant material^10^. Fukunishi et al. recently reported on inter-species differences in the resorption rates of nanofibrous vascular grafts, with a faster graft resorption of the grafts in sheep compared to rat over a 6-month timeframe. In addition, higher levels of ECM, elastin, and mature collagen were found in sheep^40^, which suggests that inter-species differences in the rates of graft resorption and tissue formation may have contributed to the differences in patency rates as observed in the present study.

### Intraspecies characteristics

In addition to inter-species differences, we analyzed model-specific, intra-species characteristics (i.e., follow-up time, animal age, and sex) via subgroup analyses. These model-specific characteristics dominantly influence the inflammatory state and intrinsic regenerative capacity, and are thereby anticipated to affect in situ tissue engineering outcome (see review De Kort and Koch et al.)^41^. The overall patency rate was dependent on follow-up time, with a significantly higher mean patency for grafts with a long follow-up time when compared to short and medium follow-up times. This suggests that occlusion is most likely to occur within short- to medium follow-up time, and is less likely to occur after a certain period of implantation. Possibly, occlusion due to thrombus mainly affects animals during the earlier stages after implantation. The influence of attrition bias is expected to be low, as the follow-up time for each animal is defined at the start of the experiment^42^. Another contributing factor may be the temporal course of the in situ remodeling processes, which is more prone to adverse remodeling at earlier stages when graft resorption and tissue deposition are in a highly active state, but much more stable once a state of tissue homeostasis has been achieved. An illustrative example is the study by Drews et al., who reported on the spontaneous reversal of early stenosis after 6 months of implantation through an inflammation-driven mechano-mediated mechanism for TEVGs that were implanted as Fontan conduit to connect the inferior vena cava and the pulmonary artery in sheep^43^.

To assess the impact of recipient age, we divided the animals in a young and an adult category, dependent on the species. Based on the studies that could be included in this meta-analysis we found that recipient age may bias graft patency rates, although the number of experimental groups in the ‘young’ category (21 experimental groups) was substantially lower than the ‘adult’ category (219 experimental groups). Very little studies have focused on understanding vascular TE with respect to recipient age^7^. It is generally assumed that young adults, here included in the adult age category, represent an optimal age due to a high regeneration capacity and relative somatic growth stability^12^. In the field of in situ TEVG, hardly any study has been performed on older/aged animals. Of all included animals, only one study reported on ‘old’ animals (*n* = 20)^26^. Aging is a risk factor for cardiovascular diseases, due to changes in vascular biology associated with age, such as arterial stiffening and cellular senescence. Moreover, aging affects the host inflammatory response to an implanted biomaterial, as has been reported for subcutaneously implanted biomaterials^44,45^. A recent study by Johnson et al. (2021) showed that PCL-gelatin fiber grafts in younger rats had less flow disturbance and healed with more organized ECM structures when compared to aged rats^46^. Considering that elderly patients are an important clinical target cohort for application TEVGs, in particular for small-diameter artery replacements, the observed influence of animal age on TEVG patency emphasizes the importance of considering animal age when evaluating in situ TEVGs. Of note, additional effects of animal size and weight were not taken into account within this analysis due to poor reporting. Younger animals, which are intrinsically smaller than adult animals, might receive grafts with smaller diameter, which is suggested to influence patency rates as well, especially for small animal models. However, species-dependent sensitivity analysis on the impact of animal age (young or adult) indicated no differences with the exclusion of any of the six species.

Next to age, there are differences in the manifestation of CVD between male and female patients. Sex-dependent differences within the cardiovascular system in general^47–49^ and in outcome after treatment with a cardiovascular prosthesis are known^50–52^. In our subgroup analysis on sex, we found no significant differences in patency rates of in situ TEVGs between male and female animals. It is plausible that the effect of sex on the in situ regenerative response is more subtle, and is not directly reflected by patency rates. For example, Blum et al. observed lower cellularity, less collagen deposition and maturation, but higher graft resorption rates in PGA + PCLA grafts when implanted as IVC interposition grafts in male mice, compared to female mice^14^. Another consideration is that for females in particular, cardiovascular pathophysiology is closely related to age, as the high estrogen levels in pre-menopausal women have a cardioprotective effect, which is lost after menopause^53^. Traditional animal models typically do not reflect such estrogen-dependent effects due to differences in hormonal regulation between species, and require more dedicated models, such as ovariectomy models^54^. Even though cardiovascular research has classically been male-oriented, awareness of sex and gender differences in the timing of diagnosis, the disease process, as well as the disease presentation is increasing over the last years^55,56^. The importance of at least reporting animal sex, let alone include both male and female animals to address the sex-specific bias, has already been described in earlier reviews on TEVGs, e.g., by Bergqvist and Jensen (1985)^57^.

### Hemodynamics and graft characteristics

Next to intrinsic animal-specific biological variables, patency is determined by the hemodynamic conditions, which are determined by the blood flow and blood pressure, as well as physical graft characteristics including graft diameter, length, and wall thickness. Therefore, we subcategorized the experimental groups into low-pressure venous and high-pressure arterial groups, further subdivided by small (<6 mm) and large (>6 mm) diameter. Interestingly, despite the often described low patency rates for small diameter blood vessels^17,27–29,58^, our results did not show a significant influence of the implant site (arterial, venous) and inner diameter (small < 6 mm < large) on the overall patency rate. This may be explained by the fact that most tested grafts are relatively short in length. In rodent models specifically, rapid transanastomotic endothelialization may favor patency rates in short grafts, while this does not occur in humans, as emphasized by Zilla et al.^17^. Clinically, small-diameter by-pass grafts have a typical length exceeding 20 cm, pre-clinical assessment of short small-diameter grafts might falsely suggest a higher patency rate, as also described by Skovrind et al.^34^. Therefore, especially for small-diameter grafts, a graft length/diameter ratio of 10 was proposed to reduce false-positive patency rates^27–29^. This threshold was only met in few studies (39/182) within this dataset.

Several groups have performed preclinical experiments to directly assess the influence of implantation site^59^. One example is a recent study by Sologashvili et al. (2019), in which PCL grafts were implanted in both the carotid and the aortic position in rats. Even though the carotid grafts showed better endothelialization, cellular infiltration, and compliance, as well as lower calcification rates compared to the aortic grafts, overall patency was only 65% in the carotid, versus 100% in the aortic grafts^15^, which is in line with our results of the subgroup analysis on implant site. The authors suggested that this difference in patency may come from differences in anatomical position, compliance mismatch, and flow conditions^15^. Indeed, the local hemodynamic conditions have been reported to play a key role for the functional remodeling of in situ TEVGs^43^, as well as thrombogenicity^60^, emphasizing the importance of the implant site when setting up animal experiments and interpreting the results thereof.

Although not specifically included in the present meta-analysis, other graft characteristics, such as wall thickness and compliance, will most certainly influence graft patency rates as well. As shown in our mapping analysis there is a great variety in synthetic materials used (Fig. 6), as well as physical properties of the graft, especially graft wall thickness and length show a large variability (Fig. 5). Given this large variability, we did not systematically analyze the effect of these graft properties on patency in the current study.

### Recommendations for animal study design and reporting

A striking observation is that for 76% of all included animals (148 studies), no health or immune status was reported. Despite the publication of the ARRIVE (Animals in Research: Reporting In Vivo Experiments) guidelines, first published in 2010 to improve reporting quality of preclinical experiments^61^, no clear improvement in reporting of health status can be observed in the studies that we assessed. Of all 148 studies that did not report on health/immune status of the animals, 59 studies were published before 2010, and 89 studies after 2010. A similar alarming conclusion was made in a systematic evaluation on reporting quality of the assessment of pulmonary heart valve prosthesis in large animals^62^. The authors concluded that the introduction of the ARRIVE guidelines did not improve reporting quality over the last 20 years in the field of heart valve research^62^. Of note, reporting of animal sex in the studies included in our review was much better after 2010, even though animal sex was not reported for 25% of all included animals (32% of all included studies). Only four studies published after 2010 did not report animal sex. Overall, we strongly recommend improved reporting of animal characteristics, following the ARRIVE guidelines.

Our findings emphasize the importance of adequate selection of the animal model and study characteristics when evaluating in situ TEVGs in a clinically relevant manner. While large animals are generally considered to better resemble the clinical situation in terms of hemodynamics and hematological profiles, small animals are easily available, require low handling and housing efforts, and are relatively affordable in larger scale cohorts^37,63,64^. Therefore, small-animal models are most often used to initially screen potential TEVGs designs and to perform early functional tests. One important observation is that the choice of animal model tends to come with a bias in other study characteristics. For example, our analyses show that, especially for small animals, implantation site is heavily biased by the choice of animal, with a clear dominance for the abdominal aorta as the preferred implantation site in rats (70% of all experiments involving rats) and rabbits (65%), and the vena cava in mice studies (70%). Additionally, almost all included male animals were rats (95%), and a vast majority of all included female animals were mice (68%). Additionally, subcategorization of age showed that the ‘young’ group was mainly derived from pigs (52% animals included in this subgroup were pigs) and sheep (20% of the total young animals). These biases are typically due to model-specific practical limitations (e.g., animal housing, surgical limitations, earlier investment in specific surgery, and specific animal model), rather than clinical relevance. In order to derive translationally meaningful data, the results of animal models should be interpreted in their appropriate context and therefore we recommend that rather than based on practical limitations, the choice of implant model should be based on clinical relevance and the proposed research question. Dedicated animal models can be applied to study specific aspects of the biological mechanisms underlying in situ vascular tissue regeneration. For example, models that have previously been described to shield transanastomotic cell ingrowth^65,66^ or animal models to mimic a multifactorial disease profile, such as vascular replacement in diabetic conditions^67^. The patient population requiring vascular replacements will mainly be the adult and elderly patient, often suffering from multifactorial diseases affecting the immunological state and regenerative capacities. Hence, we recommend to shift the scaffold-centered focus to a more graft-independent, host-dependent focus^16^. In our review, only a limited number of studies using aged and diseased animals were found, highlighting that this is a knowledge gap in the field. Even though aged and diseased animals involve more practical difficulties as well as higher costs, testing in situ TEVGs in clinically relevant animal models is highly important for clinical translation^7^. In accordance with the 3Rs (reduce, refine, replace)-perspective^68^, we advise proper design and reporting of animal studies which is essential in order to maximize the value and impact of animal studies. Especially, reporting of non-significant results should be encouraged to limit publication bias. Unfortunately, within this study, it was not possible to determine this bias due to the single arm nature of study designs and the event rate as primary measure for meta-analysis. Additionally, complementing in vivo studies with hypothesis-driven human in vitro models^69,70^ and in silico models^71,72^ can be a powerful strategy to maximize impact and accelerate translation by improving the fundamental understanding of clinically relevant processes underlying in situ vascular tissue regeneration.

### Study limitations

It is important to note that the present analysis is restricted to the collected data and to the chosen statistical evaluation methods. Despite the relatively large yield of papers, still five relevant articles^20,73–75^ were not retrieved via the search string, but found later via reference lists of relevant reviews^7,10,34,57,76–78^. Most likely this was because these five articles were not indexed at the time of retrieving the article, highlighting the effectiveness of the search string. We therefore cannot guarantee that a few eligible studies have been undetected by our search strategy. Furthermore, we only included and analyzed synthetic-based, off-the-shelf available TEVGs. We thereby exclude a wide variety of less and more successful (in situ) TEVG approaches, for example, involving decellularization of biological materials^79^, ‘bio-tubes’^80^ and pre-culturing in dedicated bioreactors^81^, for which different biological mechanisms might be involved (e.g., less dependent on inflammatory response, different thrombogenic potential). It should be noted that this distinction between natural materials and resorbable synthetic materials can be hard to establish, for example in the case of hybrid materials like Hyaff-11^82^, which is of natural origin (and thus excluded from the present study) but heavily chemically processed.

Finally, we only performed meta-analyses on patency, as the primary outcome parameter. While patency represents the functionally most important readout for a vascular graft, analyzing patency as a standalone parameter does not allow for more in-depth analysis of species-specific biological processes. The current meta-analysis was primarily intended to reveal any potential biases in outcome (in this case patency) *independent* of more specific graft design features, such as microstructural or biochemical design, which would be highly interesting in its own right. We consider the database that we built for this systematic review as a starting point for a wider variety of research questions for the field of in situ vascular TE. Further (meta-)analysis of other readout parameters, such as cellularity, ECM formation, calcification, or endothelialization, is topic of active investigation, although this is highly challenging due to large variations in analysis methods and reporting methodologies. Heterogeneity in study design, reliability of patency assessment methods, and poor reporting, might reduce the strength of the meta-analysis, as the quality of the meta-analysis relies on the quality of the primary studies. For example, we had to exclude some important subgroup categories from the meta-analysis, such as recipient old-age, due to limited number of experimental groups. This again stresses the need for more clinically relevant models.

## Conclusion

This study shows a surge in animal studies to assess the in vivo performance of resorbable synthetic vascular grafts over the past decade. Specific characteristics of animal studies, including animal species and age, as well as implantation site and follow-up time significantly affect outcome for in situ TEVGs. Several of these characteristics are biased by the choice of animal model. Additionally, health and immune-status are poorly reported on, while reporting of sex has improved over the last decade. These findings highlight the importance of adequate design of animal experiments for the testing of in situ TEVGs and proper reporting thereof in accordance to the ARRIVE guidelines, in order to maximize the value and impact of animal studies for clinical translation.

## Methods

### Review protocol

The review protocol was specified in advance and registered in an international database (PROSPERO, registration number CRD42019126716, https://www.crd.york.ac.uk/prospero/display_record.php?RecordID=126716) A few amendments to this review protocol were made and submitted to the database accordingly. The amendments involved stricter inclusion criteria for in situ TEVGs; including only primarily synthetic and off the shelf available vascular grafts. Due to limited time and resources, the hand search for additional articles was performed based on a selection of relevant reviews instead of on the references of all 182 included articles.

### Search strategy and selection of articles

PubMed and Embase (via OvidSP) were searched to identify all original articles concerning in situ TEVG in animals experiments up until July 29th 2020. The search strategy included three main components: ‘*blood vessel prosthesis*’, *‘tissue engineering*’ and ‘*animal model*’ and was composed of both indexed subject headings (MeSH and EMtree terms in PubMed and Embase) and related free text terms (for full strategies see Supplementary Tables 1 and 2). The SYRCLE animal filters were used to identify all animal studies were used, which were slightly adapted to include baboons^83,84^. No database limits were imposed, e.g., language or publication date restriction. All retrieved studies were imported to Mendeley Desktop (version 1.19.8 for Windows, Elsevier, London, UK) and duplicates were removed. Lastly, the references of a selection of relevant reviews^7,10,34,57,76–78^ were screened for potentially relevant articles that were not retrieved by the bibliographic search.

Retrieved references were screened against the inclusion criteria by two researchers per reference independently (S.K., B.K., and A.S.). First title and abstract (TIAB) screening was performed using Early Review Organizing Software Version 2.0 (EROS, Institute for Clinical Effectiveness and Health Policy, Argentina). Because the EROS website was discontinued and no longer supported during the conduct of this systematic review, the most recent TIAB screening was performed with Rayyan (www.rayyan.ai, Qatar Computing Research Institute (QCRI), Doha, Qatar). Secondly, full-text screening was performed by two researchers per reference independently (S.K., B.K., and H.B.). Any disagreements regarding the inclusion or exclusion of a particular publication were resolved by discussion. Only primary studies were included of which the study design fulfilled all of the following criteria: (1) usage of an animal model, excluding clinical trials, in silico and in vitro research, (2) usage of a vascular interposition graft, excluding portal veins, subcutaneous implantation, patches, endovascular stents, and (3) usage of an in situ TE approach. The latter was defined as a graft that is fully degradable, off-the-shelf available, and for which no donor material is necessary for production, thereby excluding long term pre-cultures in vitro (bioreactor) and in vivo (e.g., ‘biotube’ approaches).

During full-text screening, the criteria for in situ TE were more strictly defined. Only grafts produced from synthetic materials were included, thereby excluding e.g., silk, collagen, or hyaluronan-based grafts. However, for post-processing such as biofunctionalization, biological material was allowed (e.g., heparin functionalization). Additionally, for polyurethane (PU) materials non-degradability was assumed when degradability was not described nor chemical structure given. In case of doubt about the degradability of the synthetic material, chemist P.D. was consulted. Additionally, only full text articles with original data that were published in English were included.

### Quality assessment

Due to the nonrandomized, noncontrolled nature of most preclinical studies in the field of vascular tissue engineering, no standard risk of bias analysis could be performed, as validated tools are unavailable for these types of studies^85^. Instead, the quality of study design, e.g., randomization, blinding (Q2, Q3, and Q5 are based on Hooijmans et al.^85^) and analysis of multiple locations within the graft during analysis, was investigated when multiple experimental groups were compared within a study. Additionally, overall quality of reporting was scored based on the reporting of specific key information regarding (1) animal characteristics; (2) experimental setup; (3) procedure; (4) study outcome (Fig. 2)^86^. All questions were answered with ‘Yes’, ‘No’, ‘Unclear’ or ‘Not Applicable’. Detailed description of the application and meaning of these answers is reported in Supplementary Table 4. Both quality of study design and reporting was assessed independently by two researchers per study (S.K., B.K., and H.B.) and discrepancies were discussed until agreement was reached or, if no agreement could be reached, the final decision was made by a third reviewer (D.V. and A.S.).

### Data extraction

From all included studies, study characteristics, graft safety, and primary outcome measures were extracted by one independent researcher (S.K., B.K., and N.H.) and checked by a second researcher (S.K. and B.K.). Data on study characteristics included; (1) publication information (author, year, journal, DOI); (2) study characteristics (experimental groups, number of animals per group, follow-up time, graft information (i.e., material, processing technique, sterilization, storage, dimensions, compliance, biofunctionalization, shielding)); (3) animal model characteristics (animal species, animal strain, sex, age, weight, immune status, disease induction (if applicable)) and (4) intervention characteristics (implantation site, anaesthesia, analgesia, anti-coagulation, ischemia time). To report graft safety, graft-related and non-graft-related drop-outs within studies were recorded. Lastly, clinically relevant outcome measures were extracted: (1) patency, (2) thrombus formation, (3) intimal hyperplasia, (4) calcification, (5) aneurysm formation. When data were only presented graphically, a digital ruler (WebPlotDigitizer, version 4.3, https://automeris.io/WebPlotDigitizer/, Pacifica, California, USA) was used to retrieve data. Where possible, input data were converted to SI units, i.e., Graft length (mm), Graft diameter (mm) and wall thickness (µm), follow up time (days).

### Subgroup categorization

For further data mapping and meta-analysis, only relevant experimental groups in which the in situ TE approach was applicable were used, excluding the non in situ TE control groups. In order to create relevant subgroups, subcategorization of animal baseline characteristics was performed for animal age (Table 1). Due to strong variability in general life expectancy between animal species, subgroups “Young” and “Adult” were specified per animal species and defined as pre- and post-puberty^22^ respectively, including the period of puberty in the adult age group. If the text mentioned an age-category (e.g., “juvenile” or “adult” or “lamb”) the experimental group was allocated to the corresponding age-category. Subsequently, follow-up time was categorized per species, dependent on average life expectancy, with cut-off values for short vs medium and medium vs long follow-up time at 1.67 and 6.6% of total life expectancy, respectively (Table 1). Multiple locations were used as implantation site of the TEVGs, which in part also determines the graft diameter. Therefore, subcategories were defined for the high pressure application (“Arterial”) and low pressure application (“Venous”), which both were further divided based on graft diameter defining “Small diameter” as <6 mm and “Large diameter” >6 mm.

### Meta-analysis

Patency was defined as the primary outcome for meta-analysis, as this is the most important functional outcome and therefore most reported. Patency rate, and the effects of animal species, sex, age, implantation site and graft diameter, and follow-up time on patency (dataset in Supplementary Data 3) were analyzed through meta-analysis in the program Comprehensive Meta-analysis (CMA, version 3.3.070, Englewood, NJ, USA). Studies with incomplete reporting on the total number of animals used and/or number of animals with or without patency were excluded from the meta-analysis. For subgroup analysis, studies with incomplete reporting on sex, age, and follow-up time were excluded from the respective analyses. When possible, patency per follow-up time was used. However, when this was not clearly stated, the total patency rate on the latest follow-up time was used. When experimental groups contained only one allocated animal, either groups with similar experimental setup but different follow-up times within the same study were combined or, when this was not possible, the study was excluded from the analysis. Individual study data were represented by patency event rates, calculated as event rate = (e ^ LogitEventRate)/(e ^ LogitEventRate + 1), with LogitEventRate = Log(*p*/(1 – *p*)) and *p* = events/total sample size. In case of no events, 0.5 was added to event and non-event values. A random effects model was chosen to combine results, due to the diversity in animal studies^87^. I^2^ was used to express statistical heterogeneity of studies and subgroups. Grouped effects were compared through predefined subgroup analysis for animal species, sex, and implantation site. Additional post hoc subgroup analysis for animal age and follow-up time was performed. Subgroups were only included in the analysis if they contained > 10 experimental groups.

### Statistics

All original data input for the mapping figures, assessment of quality, and meta-analysis are available in the Supplementary Data 1, 2, and 3, respectively. Mapping data for graft characteristics (i.e., graft length, graft diameter, and wall thickness) are presented as mean ± standard deviation in the results section. Meta-analysis data is plotted in bar graphs with effect-size and 95% confidence interval. Statistical significance of the differences between subgroups was tested by calculation of *p*-values and correction with the conservative Bonferroni method (*p*-value*n comparisons). Significant differences (*p* < 0.05) are marked with an asterisk in the graphs. Sensitivity analysis on subgroup meta-analysis was performed for the merging of experimental groups as well as the definition of the species-dependent categorizations. Further data computation and representation were done with GraphPad PRISM (version 8.0.2 for Windows, San Diego, California, USA).

## Supplementary information

Supplemental Information

Supplemental Data 1

Supplemental Data 2

Supplemental Data 3

## Data Availability

The authors declare that the data supporting the findings of this study are available within the paper and its supplementary information files.
