# Supplementary material for: Animal studies for the evaluation of in situ tissue-engineered vascular grafts — a systematic review, evidence map, and meta-analysis
Source: NPJ Regen Med. 2022 Feb 23;7:17. doi: 10.1038/s41536-022-00211-0 (PMC8866508; doi:10.1038/s41536-022-00211-0)
Supplement: Supplementary file 1 — Supplemental Information [file 41536_2022_211_MOESM1_ESM.pdf]

Supplementary Information to:

Animal Studies for the Evaluation of *In Situ* Tissue-Engineered Vascular Grafts - a Systematic Review, Evidence Map and Meta-Analysis

Authors: Suzanne E. Koch[1,2#], Bente J. de Kort[1,2#], Noud Holshuijsen[1,2], Hannah F. M. Brouwer[1,2], Dewy C. van der Valk[1,2], Patricia Y.W. Dankers[1,2], Judith A.K.R. van Luijk[3], Carlijn R. Hooijmans[3], Rob B.M. de Vries[3], Carlijn V.C. Bouten[1,2], Anthal I.P.M. Smits[1,2 *]

Affiliations:

[1] Department of Biomedical Engineering, Eindhoven University of Technology, Eindhoven, The Netherlands

[2] Institute for Complex Molecular Systems (ICMS), Eindhoven University of Technology, Eindhoven, The Netherlands

[3] SYstematic Review Centre for Laboratory animal Experimentation (SYRCLE), Department for Health Evidence, Radboud Institute for Health Sciences, Radboud UMC, Nijmegen, The Netherlands

[#] SK and BK contributed equally

*** Corresponding author:**

Anthal I.P.M. Smits

Eindhoven University of Technology, Department of Biomedical Engineering;

PO Box 513, 5600 MB Eindhoven, The Netherlands;

Email: a.i.p.m.smits@tue.nl; Tel: +31 40 247 4738

Supplementary Table 1: Search strategy for Pubmed using search string defining the components "blood vessel prosthesis", "tissue engineering" and "animal"

Component	Search entry
Component 1: blood vessel prosthesis	"Blood Vessel Prosthesis"[MeSH] OR (((artery [tiab] OR arteries [tiab] OR vein [tiab] OR veins [tiab] OR vascular [tiab] OR arterial [tiab] OR venous [tiab]) AND (prostheses [tiab] OR prosthesis [tiab] OR graft [tiab] OR grafts [tiab] OR scaffold [tiab] OR scaffolds [tiab] OR replacement [tiab] OR replacements [tiab] OR substitute [tiab] OR substitutes [tiab] OR grafting [tiab])
Component 2: tissue engineering	AND ("tissue engineering" [tiab] OR "tissue engineered" [tiab] OR "Tissue Engineering" [MeSH Terms] OR "Guided Tissue Regeneration" [MeSH] OR regeneration [tiab]))) OR "TEVG"[tiab] OR ((artery [tiab] OR arteries [tiab] OR vein [tiab] OR veins [tiab] OR vascular [tiab] OR arterial [tiab] OR venous [tiab]) AND ("tissue engineering" [tiab] OR "tissue engineered" [tiab] OR "Tissue Engineering" [MeSH Terms] OR "Guided Tissue Regeneration" [MeSH])) OR "Tissue engineered blood vessel" [tiab] OR "Tissue engineered blood vessels" [tiab] OR "Blood vessel tissue engineering" [tiab] OR "Blood vessel regeneration" [tiab] OR (((degradable[tiab] OR biodegradable[tiab] OR resorbable[tiab] OR bioresorbable[tiab] OR regenerative[tiab]) AND ("blood vessel"[tiab] OR artery [tiab] OR arteries [tiab] OR vein [tiab] OR veins [tiab] OR vascular [tiab] OR arterial [tiab] OR venous [tiab]) AND (prostheses [tiab] OR prosthesis [tiab] OR graft [tiab] OR grafts [tiab] OR scaffold [tiab] OR scaffolds [tiab] OR replacement [tiab] OR replacements [tiab] OR substitute [tiab] OR substitutes [tiab])))
Component 3: animal (adapted version of[1,2])	("animal experimentation"[MeSH Terms] OR "models, animal"[MeSH Terms] OR "invertebrates"[MeSH Terms] OR "Animals"[Mesh:noexp] OR "animal population groups"[MeSH Terms] OR "chordata"[MeSH Terms:noexp] OR "chordata, nonvertebrate"[MeSH Terms] OR "vertebrates"[MeSH Terms:noexp] OR "amphibians"[MeSH Terms] OR "birds"[MeSH Terms] OR "fishes"[MeSH Terms] OR "reptiles"[MeSH Terms] OR "mammals"[MeSH Terms:noexp] OR "primates"[MeSH Terms:noexp] OR "artiodactyla"[MeSH Terms] OR "carnivora"[MeSH Terms] OR "cetacea"[MeSH Terms] OR "chiroptera"[MeSH Terms] OR "elephants"[MeSH Terms] OR "hyraxes"[MeSH Terms] OR "insectivora"[MeSH Terms] OR "lagomorpha"[MeSH Terms] OR "marsupialia"[MeSH Terms] OR "monotremata"[MeSH Terms] OR "perissodactyla"[MeSH Terms] OR "rodentia"[MeSH Terms] OR "scandentia"[MeSH Terms] OR "sirenia"[MeSH Terms] OR "xenarthra"[MeSH Terms] OR "haplorhini"[MeSH Terms:noexp] OR "strepsirhini"[MeSH Terms] OR "platyrrhini"[MeSH Terms] OR "tarsii"[MeSH Terms] OR "catarrhini"[MeSH Terms:noexp] OR "cercopithecidae"[MeSH Terms] OR "hylobatidae"[MeSH Terms] OR "hominidae"[MeSH Terms:noexp] OR "gorilla gorilla"[MeSH Terms] OR "pan paniscus"[MeSH Terms] OR "pan troglodytes"[MeSH Terms] OR "pongo pygmaeus"[MeSH Terms]) OR ((animals[tiab] OR animal[tiab] OR mice[Tiab] OR mus[Tiab] OR mouse[Tiab] OR murine[Tiab] OR woodmouse[tiab] OR rats[Tiab] OR rat[Tiab] OR murinae[Tiab] OR muridae[Tiab] OR cottonrat[tiab] OR cottonrats[tiab] OR hamster[tiab] OR hamsters[tiab] OR cricetinae[tiab] OR rodentia[Tiab] OR rodent[Tiab] OR rodents[Tiab] OR pigs[Tiab] OR pig[Tiab] OR swine[tiab] OR swines[tiab] OR piglets[tiab] OR piglet[tiab] OR boar[tiab] OR boars[tiab] OR "sus scrofa"[tiab] OR ferrets[tiab] OR ferret[tiab] OR polecat[tiab] OR polecats[tiab] OR "mustela putorius"[tiab] OR "guinea pigs"[Tiab] OR "guinea pig"[Tiab] OR cavia[Tiab] OR callithrix[Tiab] OR marmoset[Tiab] OR marmosets[Tiab] OR cebuella[Tiab] OR hapale[Tiab] OR octodon[Tiab] OR chinchilla[Tiab] OR chinchillas[Tiab] OR gerbillinae[Tiab] OR gerbil[Tiab] OR gerbils[Tiab] OR jird[Tiab] OR jirds[Tiab] OR merione[Tiab] OR meriones[Tiab] OR rabbits[Tiab] OR rabbit[Tiab] OR hares[Tiab] OR hare[Tiab] OR diptera[Tiab] OR flies[Tiab] OR fly[Tiab] OR dipteral[Tiab] OR drosophila[Tiab] OR drosophilidae[Tiab] OR cats[Tiab] OR cat[Tiab] OR carus[Tiab] OR felis[Tiab] OR nematoda[Tiab] OR nematode[Tiab] OR nematodes[Tiab] OR sipunculida[Tiab] OR dogs[Tiab] OR dog[Tiab] OR canine[Tiab] OR canines[Tiab] OR canis[Tiab] OR sheep[Tiab] OR sheeps[Tiab] OR mouflon[Tiab] OR mouflons[Tiab] OR ovis[Tiab] OR goats[Tiab] OR goat[Tiab] OR capra[Tiab] OR capras[Tiab] OR rupicapra[Tiab] OR rupicapras[Tiab] OR chamois[Tiab] OR haplorhini[Tiab] OR monkey[Tiab] OR monkeys[Tiab] OR anthropoidea[Tiab] OR anthropoids[Tiab] OR saguinus[Tiab] OR tamarin[Tiab] OR tamarins[Tiab] OR leontopithecus[Tiab] OR hominidae[Tiab] OR ape[Tiab] OR apes[Tiab] OR "pan paniscus"[Tiab] OR bonobo[Tiab] OR bonobos[Tiab] OR "pan troglodytes"[Tiab] OR gibbon[Tiab] OR gibbons[Tiab] OR siamang[Tiab] OR siamangs[Tiab] OR nomascus[Tiab] OR symphalangus[Tiab] OR chimpanzee[Tiab] OR chimpanzees[Tiab] OR prosimian[Tiab] OR prosimians[Tiab] OR "bush baby"[Tiab] OR bush babies[Tiab] OR galagos[Tiab] OR galago[Tiab] OR pongidae[Tiab] OR gorilla[Tiab] OR gorillas[Tiab] OR "pongo pygmaeus"[Tiab] OR orangutan[Tiab] OR orangutans[Tiab] OR lemur[Tiab] OR

	lemurs[Tiab] OR lemuridae[Tiab] OR horse[Tiab] OR horses[Tiab] OR equus[Tiab] OR cow[Tiab] OR calf[Tiab] OR bull[Tiab] OR chicken[Tiab] OR chickens[Tiab] OR gallus[Tiab] OR quail[Tiab] OR bird[Tiab] OR birds[Tiab] OR quails[Tiab] OR poultry[Tiab] OR poultries[Tiab] OR fowl[Tiab] OR fowls[Tiab] OR reptile[Tiab] OR reptilia[Tiab] OR reptiles[Tiab] OR snakes[Tiab] OR snake[Tiab] OR lizard[Tiab] OR lizards[Tiab] OR alligator[Tiab] OR alligators[Tiab] OR crocodile[Tiab] OR crocodiles[Tiab] OR turtle[Tiab] OR turtles[Tiab] OR amphibian[Tiab] OR amphibians[Tiab] OR amphibia[Tiab] OR frog[Tiab] OR frogs[Tiab] OR bombina[Tiab] OR salientia[Tiab] OR toad[Tiab] OR toads[Tiab] OR “epidalea calamita”[Tiab] OR salamander[Tiab] OR salamanders[Tiab] OR eel[Tiab] OR eels[Tiab] OR fish[Tiab] OR fishes[Tiab] OR pisces[Tiab] OR catfish[Tiab] OR catfishes[Tiab] OR siluriformes[Tiab] OR arius[Tiab] OR heteropneustes[Tiab] OR sheatfish[Tiab] OR perch[Tiab] OR perches[Tiab] OR percidae[Tiab] OR perca[Tiab] OR trout[Tiab] OR trouts[Tiab] OR char[Tiab] OR chars[Tiab] OR salvelinus[Tiab] OR minnow[Tiab] OR cyprinidae[Tiab] OR carps[Tiab] OR carp[Tiab] OR zebrafish[Tiab] OR zebrafishes[Tiab] OR goldfish[Tiab] OR goldfishes[Tiab] OR guppy[Tiab] OR guppies[Tiab] OR chub[Tiab] OR chubs[Tiab] OR tinca[Tiab] OR barbels[Tiab] OR barbus[Tiab] OR pimephales[Tiab] OR promelas[Tiab] OR “poecilia reticulata”[Tiab] OR mullet[Tiab] OR mullets[Tiab] OR eel[Tiab] OR eels[Tiab] OR seahorse[Tiab] OR seahorses[Tiab] OR mugil curema[Tiab] OR atlantic cod[Tiab] OR shark[Tiab] OR sharks[Tiab] OR catshark[Tiab] OR anguilla[Tiab] OR salmonid[Tiab] OR salmonids[Tiab] OR whitefish[Tiab] OR whitefishes[Tiab] OR salmon[Tiab] OR salmons[Tiab] OR sole[Tiab] OR solea[Tiab] OR lamprey[Tiab] OR lampreys[Tiab] OR pumpkinseed[Tiab] OR sunfish[Tiab] OR sunfishes[Tiab] OR tilapia[Tiab] OR tilapias[Tiab] OR turbot[Tiab] OR turbots[Tiab] OR flatfish[Tiab] OR flatfishes[Tiab] OR sciuridae[Tiab] OR squirrel[Tiab] OR squirrels[Tiab] OR chipmunk[Tiab] OR chipmunks[Tiab] OR suslik[Tiab] OR susliks[Tiab] OR vole[Tiab] OR voles[Tiab] OR lemming[Tiab] OR lemmings[Tiab] OR muskrat[Tiab] OR muskrats[Tiab] OR lemmus[Tiab] OR otter[Tiab] OR otters[Tiab] OR marten[Tiab] OR martens[Tiab] OR martes[Tiab] OR weasel[Tiab] OR badger[Tiab] OR badgers[Tiab] OR ermine[Tiab] OR mink[Tiab] OR minks[Tiab] OR sable[Tiab] OR sables[Tiab] OR gulo[Tiab] OR gulos[Tiab] OR wolverine[Tiab] OR wolverines[Tiab] OR mustela[Tiab] OR llama[Tiab] OR llamas[Tiab] OR alpaca[Tiab] OR alpacas[Tiab] OR camelid[Tiab] OR camelids[Tiab] OR guanaco[Tiab] OR guanacos[Tiab] OR chiroptera[Tiab] OR chiropteras[Tiab] OR bat[Tiab] OR bats[Tiab] OR fox[Tiab] OR foxes[Tiab] OR iguana[Tiab] OR iguanas[Tiab] OR xenopus laevis[Tiab] OR parakeet[Tiab] OR parakeets[Tiab] OR parrot[Tiab] OR parrots[Tiab] OR donkey[Tiab] OR donkeys[Tiab] OR mule[Tiab] OR mules[Tiab] OR zebra[Tiab] OR zebras[Tiab] OR shrew[Tiab] OR shrews[Tiab] OR bison[Tiab] OR bisons[Tiab] OR buffalo[Tiab] OR buffaloes[Tiab] OR deer[Tiab] OR deers[Tiab] OR bear[Tiab] OR bears[Tiab] OR panda[Tiab] OR pandas[Tiab] OR “wild hog”[Tiab] OR “wild boar”[Tiab] OR fitchew[Tiab] OR fitch[Tiab] OR beaver[Tiab] OR beavers[Tiab] OR jerboa[Tiab] OR jerboas[Tiab] OR capybara[Tiab] OR capybaras[Tiab] OR canine [tiab] OR bovine [tiab] OR porcine [tiab] OR hog [tiab] OR hogs [tiab]) OR baboon[tiab] OR baboons[tiab] OR non-human primate[tiab] OR non-human primates[tiab] OR non human primate[tiab] OR non human primates [tiab] OR monkey[tiab] OR monkeys[tiab]

Supplementary Table 2: Search strategy for Embase using search string defining the components "blood vessel prosthesis", "tissue engineering" and "animal"

**Component**	**Search entry**
Component 1: blood vessel prosthesis	exp Blood Vessel Prosthesis/ or (((artery or arteries or vein or veins or vascular or arterial or venous) and (prostheses or prosthesis or graft or grafts or scaffold or scaffolds or replacement or replacements or substitute or substitutes or grafting))
Component 2: tissue engineering	ti,ab,kw. and ((tissue engineering or tissue engineered).ti,ab,kw. or exp Tissue Engineering/ or tissue regeneration/ or exp tissue repair/ or guided Tissue Regeneration.ti,ab,kw. or regeneration.ti,ab,kw.)) or TEVG.ti,ab,kw. or ((artery or arteries or vein or veins or vascular or arterial or venous).ti,ab,kw. and ((tissue engineering or tissue engineered).ti,ab,kw. or exp Tissue Engineering/ or tissue regeneration/ or exp tissue repair/ or guided Tissue Regeneration.ti,ab,kw.)) or Tissue engineered blood vessel.ti,ab,kw. or Tissue engineered blood vessels.ti,ab,kw. or Blood vessel tissue engineering.ti,ab,kw. or Blood vessel regeneration.ti,ab,kw. or ((degradable or biodegradable or resorbable or bioresorbable or regenerative) and (blood vessel or artery or arteries or vein or veins or vascular or arterial or venous) and (prostheses or prosthesis or graft or grafts or scaffold or scaffolds or replacement or replacements or substitute or substitutes)).ti,ab,kw.
Component 3: animal (adapted version of[1,2])	exp animal experiment/ or exp animal model/ or exp experimental animal/ or exp transgenic animal/ or exp male animal/ or exp female animal/ or exp juvenile animal/ or animal/ or chordata/ or vertebrate/ or tetrapod/ or exp fish/ or amniote/ or exp amphibia/ or mammal/ or exp reptile/ or exp sauropsid/ or therian/ or exp monotremate/ or placental mammals/ or exp marsupial/ or Euarchontoglires/ or exp Afrotheria/ or exp Boreoeutheria/ or exp Laurasiatheria/ or exp Xenarthra/ or primate/ or exp Dermoptera/ or exp Glires/ or exp Scandentia/ or Haplorhini/ or exp prosimian/ or simian/ or exp tarsiiform/ or Catarrhini/ or exp Platyrrhini/ or ape/ or exp Cercopithecidae/ or hominid/ or exp hylobatidae/ or exp chimpanzee/ or exp gorilla/ or exp orang utan/(animal or animals or pisces or fish or fishes or catfish or catfishes or sheatfish or silurus or arius or heteropneustes or clarias or gariepinus or fathead minnow or fathead minnows or pimephales or promelas or cichlidae or trout or trouts or char or chars or salvelinus or salmo or oncorhynchus or guppy or guppies or millionfish or poecilia or goldfish or goldfishes or carassius or auratus or mullet or mullets or mugil or curema or shark or sharks or cod or cods or gadus or morhua or carp or carps or cyprinus or carpio or killifish or eel or eels or anguilla or zander or sander or lucioperca or stizostedion or turbot or turbots or psetta or flatfish or flatfishes or plaice or pleuronectes or platessa or tilapia or tilapias or oreochromis or sarotherodon or common sole or dover sole or solea or zebrafish or zebrafishes or danio or rerio or seabass or dicentrarchus or labrax or morone or lamprey).ti,ab. (lampreys or petromyzon or pumpkinseed or pumpkinseeds or lepomis or gibbosus or herring or clupea or harengus or amphibia or amphibian or amphibians or anura or salientia or frog or frogs or rana or toad or toads or bufo or xenopus or laevis or bombina or epidalea or calamita or salamander or salamanders or newt or newts or triturus or reptilia or reptile or reptiles or bearded dragon or pogona or vitticeps or iguana or iguanas or lizard or lizards or anguis fragilis or turtle or turtles or snakes or snake or aves or bird or birds or quail or quails or coturnix or bobwhite or colinus or virginianus or poultry or poultries or fowl or fowls or chicken or chickens or gallus or zebra finch or taeniopygia or guttata or canary or canaries or serinus or canaria or parakeet or parakeets or grasskeet or parrot or parrots or psittacine or psittacines or shelduck or tadorna or goose or geese or branta or leucopsis or woodlark or lullula or flycatcher or ficedula or hypoleuca or dove or doves or geopelia or cuneata or duck or ducks or greylag or graylag or anser or harrier or circus pygargus or red knot or great knot or calidris or canutus).ti,ab. (godwit or limosa or lapponica or meleagris or gallopavo or jackdaw or corvus or monedula or ruff or philomachus or pugnax or lapwing or peewit or plover or vanellus or swan or cygnus or columbianus or bewickii or gull or chroicocephalus or ridibundus or albifrons or great tit or parus or aythya or fuligula or streptopelia or risoria or spoonbill or platalea or leucorodia or blackbird or turdus or merula or blue tit or cyanistes or pigeon or pigeons or columba or pintail or anas or starling or sturnus or owl or athene noctua or pochard or ferina or cockatiel or nymphicus or hollandicus or skylark or alauda or tern or sterna or teal or crecca or oystercatcher or haematopus or ostralegus or shrew or shrews or sorex or araneus or crocidura or russula or european mole or talpa or chiroptera or bat or bats or eptesicus or serotinus or myotis or dasycneme or daubentonii or pipistrelle or pipistrellus or cat or cats or felis or catus or feline or dog or dogs or canis or canine or canines or otter or otters or lutra or badger or badgers or meles or fitchew or fitch or foumart or foulmart or ferrets or ferret or polecat or polecats or mustela or putorius or weasel or weasels or fox or foxes or vulpes or common seal or phoca or vitulina or grey

	seal or halichoerus or horse or horses or equus or equine or equidae or donkey or donkeys or mule or mules or pig or pigs or swine or swines or hog or hogs or boar or boars or porcine or piglet or piglets or sus or scrofa or llama or llamas or lama or glama or deer or deers or cervus or elaphus or cow or cows or bos taurus or bos indicus or bovine or bull or bulls or cattle or bison or bisons or sheep or sheeps or ovis aries or ovine or lamb or lambs or mouflon or mouflons or goat or goats or capra or caprine or chamois or rupicapra or leporidae).ti,ab. (lagomorpha or lagomorph or rabbit or rabbits or oryctolagus or cuniculus or laprine or hares or lepus or rodentia or rodent or rodents or murinae or mouse or mice or mus or musculus or murine or woodmouse or apodemus or rat or rats or rattus or norvegicus or guinea pig or guinea pigs or cavia or porcellus or hamster or hamsters or mesocricetus or cricetulus or cricetus or gerbil or gerbils or jird or jirds or meriones or unguiculatus or jerboa or jerboas or jaculus or chinchilla or chinchillas or beaver or beavers or castor fiber or castor canadensis or sciuridae or squirrel or squirrels or sciurus or chipmunk or chipmunks or marmot or marmots or marmota or suslik or susliks or spermophilus or cynomys or cottonrat or cottonrats or sigmodon or vole or voles or microtus or myodes or glareolus or primate or primates or prosimian or prosimians or lemur or lemurs or lemuridae or loris or bush baby or bush babies or bushbaby or bushbabies or galago or galagos or anthropoidea or anthropoids or simian or simians or monkey or monkeys or marmoset or marmosets or callithrix or cebuella or tamarin or tamarins or saguinus or leontopithecus or squirrel monkey or squirrel monkeys or saimiri or night monkey or night monkeys or owl monkey or owl monkeys or douroucoulis or aotus or spider monkey or spider monkeys or ateles or baboon or baboons or papio or rhesus monkey or macaque or macaca or mulatta or cynomolgus or fascicularis or green monkey or green monkeys or chlorocebus or vervet or vervets or pygerythrus or hominoidea or ape or apes or hylobatidae or gibbon or gibbons or siamang or siamangs or nomascus or symphalangus or hominidae or orangutan or orangutans or pongo or chimpanzee or chimpanzees or pan troglodytes or bonobo or bonobos or pan paniscus or gorilla or gorillas or troglodytes).ti,ab. (non-human primate or non-human primates or non human primate or non human primates).ti,ab.

Supplementary Table 3: abbreviations of polymers

**Abbreviation**	**Full name**	**Examples within this category and commercial names**
CE-UPY	Chain extended- ureido-pyrimidinone	
P(BT-BLOCK-EG)	poly(polybutylene terephthalate-co-polyether glycol)	Hyrtel
P(LA-CO-GA)	poly(lactid acid-co-glycolic acid)	PHO-PGA
P(LLA-CO-CL)	poly(L-Lactic-co-caprolactone)	PLCL, PHBV/PCL, PCL2000-U4U, PPF
PA	polyamide	Nylon
PBF	poly (2, 3-butylene furmarate	
PCL	polycaprolactone	hPCL tPCL
PCU	polycarbonate urethane	dPCU(BHPC_211), reinforced Corvita
PDLA	poly-D-L-lactic acid	
PDMS	polydimethylsiloxane	
PDS	polydioxanone	
PEG	polyethylene glycol	
PETU	poly(ether)urethane	Estane
PEU	polyesterurethane	Pulsetec, poly(1-LEU-10)
PEUU	polyesterurethane urea	Mitrathane (PEUU), Thoratec
PGA	polyglycolic acid	PG10, PG910, Vicryl
PHEA	a,b-poly(N-2-hydroxyethyl)-D,L-aspartamide	
PLLA	poly-l-lactic acid	
PMEH	poly(2-methacryloyloxyethyl phosphorylcholine-co-2-Ethylhexyl methacrylate)	
PTX	Paclitaxel	
PU	polyurethane	Thermoplastic polyurethanes, segmented polyurethane (SPU), Biomer, Mitrathane (PU)
PUSN	poly(ester urethane)urea with disulfide and amino group	
PVA	polyvinyl alcohol	Ivalon sponge
SE	synthetic elastin	

Supplementary Table 4: definition of answers for quality assessment of study design and reporting of preclinical studies.

Experimental set-up	
Q1 Were there different experimental groups? Yes → continue next 2 questions. No → stop	1 experimental group = same material, same implant side, multiple time points explanted<br>No means that there was only 1 experimental group.
Q2 Was allocation of animals to experimental groups random?	Yes: when stated in the text that they were randomly assessed, or that the grafts were randomly implanted / surgeon was blinded.<br>No: when stated that that was not the case, or because of experimental design it was not random.<br>Unclear: when it was not described.<br>N.A.: when there were no different experimental groups
Q3 Was the outcome assessor blinded for the experimental groups (for any compared qualitative outcome?)	N.A.: when there were no different experimental groups<br>Unclear: when it was not stated if the assessor was blinded.<br>Yes: when it was stated that the outcome assessor was blinded.<br>No: when it was stated that the outcome assessor was not blinded
Study Outcome	
Q4 Are multiple locations within the vascular graft analyzed per animal?	Yes: when observations were compared between proximal, distal, mid-graft or anastomosis side. Or when angiogram, doppler ultra sound or complete full length staining/SEM analysis was performed<br>No: when it was stated in method section that only the mid portion was analyzed<br>Unclear: when it was not stated, nor became clear from M&M
Q5 Are explanted vascular graft analyzed randomly selected?	Yes when it was stated that the outcome assessor was blinded, or when the order of analysis of the samples was random.<br>Unclear: when it was not stated if the samples were randomly assessed.<br>NA when there was only 1 included animal.
Reporting of Animal Information	
Q6 Is the animal species described?	Yes: animal species described
Q7 Is the animal strain described?	Unclear: when it was only described for some experimental groups
Q8 Is the sex of animals described?	Unclear: when it was only described for some experimental groups
Q9 Is the age of animals described?	Unclear when only “young” or “adult” “juvenile” was given
Q10 Is the weight of animals described?	
Q11 Is the ethical review board described?	Yes: when board was given, but also when referred to NIH guidelines<br>Unclear: when only stated : “approved by a local ethic committee”
Q12 Is the number of animals allocated to each experimental group described?	Unclear: when not described in material and methods, and only mentioned in 1 figure legend
Q13 Is the allocated follow-up time described?	Unclear: when not described for all experimental groups
Reporting of Procedure:	
Q14 Is the synthetic material of the vascular graft described?	Yes: this was an inclusion criterium
Q15 Are the dimensions of the vascular graft described? (length, diameter, wall thickness)	Yes: when all 3 values were given: length, inner diameter and wall thickness<br>Unclear: when only 1/3 or 2/3 dimensions were given<br>No: when none of these were given

Q16 Is sterilization/disinfection of the vascular grafts described?	Unclear: when not described for all experimental groups / or when only mentioned “worked sterile”/ “sterilized” but no method given
Q17 Is the vascular graft storage prior to implantation described?	Unclear: when only stated “packed”
Q18 If applicable, is the procedure for functionalization of the vascular graft described?	Unclear: when it is described, but no clear method is given and no references to other papers in which it is described
Q19 If applicable, is the procedure for on-the-fly cell seeding of the vascular graft described?	Yes: described or referred to a source describing the seeding process.
Q20 Is the location of implantation of the vascular graft described?	Yes: all answered yes, because one of the inclusion criteria
Q21 Is the surgical procedure of implantation of the vascular graft described (including references to other papers)?	No: when no explanation is given and no references to other papers<br>Unclear when no reference is given and when the surgical procedure is hardly described
Q23 Is the use and period of anti-coagulation described?	Unclear: when only the use is given during operation or only post implantation, but not both<br>No: not at all stated if and when anticoagulation was used. Could also be the case for when authors refer to other source for the implantation procedure, but then not “briefly” describe the most important details like eg use of anticoagulation.
Q23 Is the use and period of anaesthesia, analgesia described?	Unclear when only was stated “animals were anesthetized”<br>No: when not reported. Could also be the case for when authors refer to other source for the implantation procedure, but then not “briefly” describe the most important details like eg anesthesia.
Reporting of general outcome:	
Q24 Are adverse events described?	Unclear: only based on occlusion rates, but not really described that they looked for eg thrombosis / stenosis / aneurysm / other complications
Q25 Is the final number of animals analyzed in each experimental group per timepoint described?	Yes: when the initial number of included animals was clear and the drop outs per group.<br>No: when the initial number of included animals were not clear Unclear: when the drop outs per timepoint were not clear, but for the overall group were clear
Q26 Are numbers of drop-outs described?	Unclear: when not given for all experimental groups. Or when not clearly stated, but only when calculating by the reader the drop outs can be known.<br>NA: when only 1 animal was included and it survived
Q26a If applicable: Are the reasons for drop-outs described?	Yes: when cause was given, NB: also when stated “death due to unknown cause”<br>No: when not given, only mentioned death.<br>NA when all animals survived and no animals dropped out<br>Unclear when animals dropped out, and you know the number, but not the reason why
Q26b If applicable: Is the timepoint of drop-outs described?	Yes: timepoint is mentioned (perioperative or the timeframe in which the animals died prior to planned explant-time)<br>No: when not given, only mentioned deaths per experimental groups.<br>NA when all animals survived and no animals dropped out<br>Unclear when animals dropped out, and you know the amount, but not the timepoint

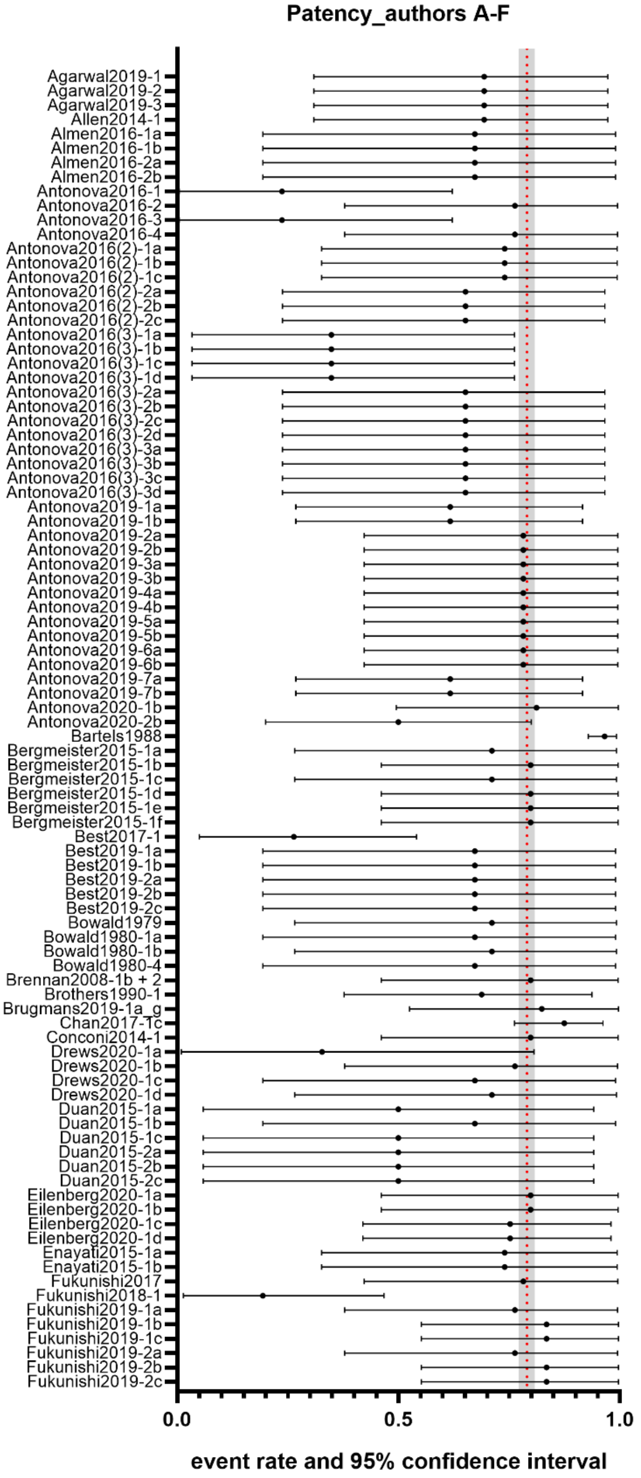
Patency_authors A-F
Agarwal2019-1
Agarwal2019-2
Agarwal2019-3
Allen2014-1
Almen2016-1a
Almen2016-1b
Almen2016-2a
Almen2016-2b
Antonova2016-1
Antonova2016-2
Antonova2016-3
Antonova2016-4
Antonova2016(2)-1a
Antonova2016(2)-1b
Antonova2016(2)-1c
Antonova2016(2)-2a
Antonova2016(2)-2b
Antonova2016(2)-2c
Antonova2016(3)-1a
Antonova2016(3)-1b
Antonova2016(3)-1c
Antonova2016(3)-1d
Antonova2016(3)-2a
Antonova2016(3)-2b
Antonova2016(3)-2c
Antonova2016(3)-2d
Antonova2016(3)-3a
Antonova2016(3)-3b
Antonova2016(3)-3c
Antonova2016(3)-3d
Antonova2019-1a
Antonova2019-1b
Antonova2019-2a
Antonova2019-2b
Antonova2019-3a
Antonova2019-3b
Antonova2019-4a
Antonova2019-4b
Antonova2019-5a
Antonova2019-5b
Antonova2019-6a
Antonova2019-6b
Antonova2019-7a
Antonova2019-7b
Antonova2020-1b
Antonova2020-2b
Bartels1988
Bergmeister2015-1a
Bergmeister2015-1b
Bergmeister2015-1c
Bergmeister2015-1d
Bergmeister2015-1e
Bergmeister2015-1f
Best2017-1
Best2019-1a
Best2019-1b
Best2019-2a
Best2019-2b
Best2019-2c
Bowald1979
Bowald1980-1a
Bowald1980-1b
Bowald1980-4
Brennan2008-1b + 2
Brothers1990-1
Brugmans2019-1a_g
Chan2017-1c
Conconi2014-1
Drews2020-1a
Drews2020-1b
Drews2020-1c
Drews2020-1d
Duan2015-1a
Duan2015-1b
Duan2015-1c
Duan2015-2a
Duan2015-2b
Duan2015-2c
Eilenberg2020-1a
Eilenberg2020-1b
Eilenberg2020-1c
Eilenberg2020-1d
Enayati2015-1a
Enayati2015-1b
Fukunishi2017
Fukunishi2018-1
Fukunishi2019-1a
Fukunishi2019-1b
Fukunishi2019-1c
Fukunishi2019-2a
Fukunishi2019-2b
Fukunishi2019-2c
0.0
0.5
1.0
event rate and 95% confidence interval

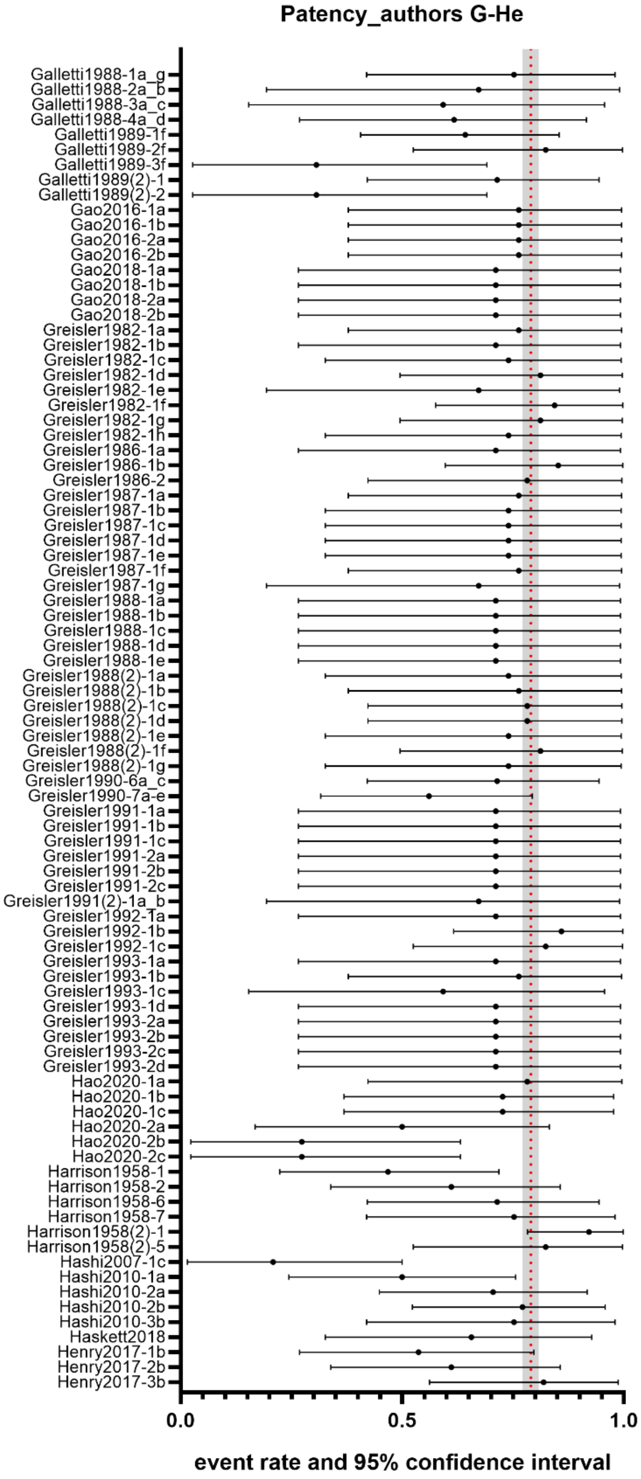
Patency_authors G-He
Galletti1988-1a_g
Galletti1988-2a_b
Galletti1988-3a_c
Galletti1988-4a_d
Galletti1989-1f
Galletti1989-2f
Galletti1989-3f
Galletti1989(2)-1
Galletti1989(2)-2
Gao2016-1a
Gao2016-1b
Gao2016-2a
Gao2016-2b
Gao2018-1a
Gao2018-1b
Gao2018-2a
Gao2018-2b
Greisler1982-1a
Greisler1982-1b
Greisler1982-1c
Greisler1982-1d
Greisler1982-1e
Greisler1982-1f
Greisler1982-1g
Greisler1982-1h
Greisler1986-1a
Greisler1986-1b
Greisler1986-2
Greisler1987-1a
Greisler1987-1b
Greisler1987-1c
Greisler1987-1d
Greisler1987-1e
Greisler1987-1f
Greisler1987-1g
Greisler1988-1a
Greisler1988-1b
Greisler1988-1c
Greisler1988-1d
Greisler1988-1e
Greisler1988(2)-1a
Greisler1988(2)-1b
Greisler1988(2)-1c
Greisler1988(2)-1d
Greisler1988(2)-1e
Greisler1988(2)-1f
Greisler1988(2)-1g
Greisler1990-6a_c
Greisler1990-7a-e
Greisler1991-1a
Greisler1991-1b
Greisler1991-1c
Greisler1991-2a
Greisler1991-2b
Greisler1991-2c
Greisler1991(2)-1a_b
Greisler1992-1a
Greisler1992-1b
Greisler1992-1c
Greisler1993-1a
Greisler1993-1b
Greisler1993-1c
Greisler1993-1d
Greisler1993-2a
Greisler1993-2b
Greisler1993-2c
Greisler1993-2d
Hao2020-1a
Hao2020-1b
Hao2020-1c
Hao2020-2a
Hao2020-2b
Hao2020-2c
Harrison1958-1
Harrison1958-2
Harrison1958-6
Harrison1958-7
Harrison1958(2)-1
Harrison1958(2)-5
Hashi2007-1c
Hashi2010-1a
Hashi2010-2a
Hashi2010-2b
Hashi2010-3b
Haskett2018
Henry2017-1b
Henry2017-2b
Henry2017-3b
0.0
0.5
1.0
event rate and 95% confidence interval

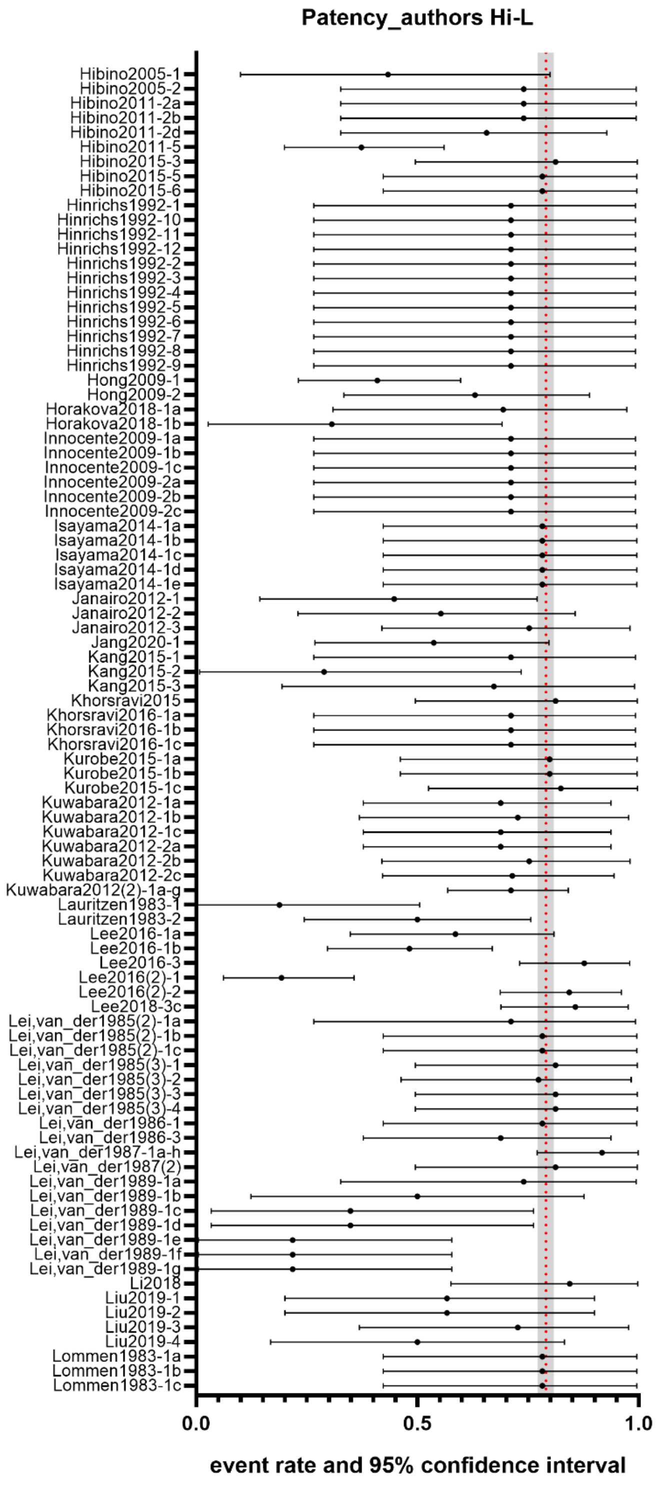
Patency_authors Hi-L
Hibino2005-1
Hibino2005-2
Hibino2011-2a
Hibino2011-2b
Hibino2011-2d
Hibino2011-5
Hibino2015-3
Hibino2015-5
Hibino2015-6
Hinrichs1992-1
Hinrichs1992-10
Hinrichs1992-11
Hinrichs1992-12
Hinrichs1992-2
Hinrichs1992-3
Hinrichs1992-4
Hinrichs1992-5
Hinrichs1992-6
Hinrichs1992-7
Hinrichs1992-8
Hinrichs1992-9
Hong2009-1
Hong2009-2
Horakova2018-1a
Horakova2018-1b
Innocente2009-1a
Innocente2009-1b
Innocente2009-1c
Innocente2009-2a
Innocente2009-2b
Innocente2009-2c
Isayama2014-1a
Isayama2014-1b
Isayama2014-1c
Isayama2014-1d
Isayama2014-1e
Janairo2012-1
Janairo2012-2
Janairo2012-3
Jang2020-1
Kang2015-1
Kang2015-2
Kang2015-3
Khorsravi2015
Khorsravi2016-1a
Khorsravi2016-1b
Khorsravi2016-1c
Kurobe2015-1a
Kurobe2015-1b
Kurobe2015-1c
Kuwabara2012-1a
Kuwabara2012-1b
Kuwabara2012-1c
Kuwabara2012-2a
Kuwabara2012-2b
Kuwabara2012-2c
Kuwabara2012(2)-1a-g
Lauritzen1983-1
Lauritzen1983-2
Lee2016-1a
Lee2016-1b
Lee2016-3
Lee2016(2)-1
Lee2016(2)-2
Lee2018-3c
Lei,van_der1985(2)-1a
Lei,van_der1985(2)-1b
Lei,van_der1985(2)-1c
Lei,van_der1985(3)-1
Lei,van_der1985(3)-2
Lei,van_der1985(3)-3
Lei,van_der1985(3)-4
Lei,van_der1986-1
Lei,van_der1986-3
Lei,van_der1987-1a-h
Lei,van_der1987(2)
Lei,van_der1989-1a
Lei,van_der1989-1b
Lei,van_der1989-1c
Lei,van_der1989-1d
Lei,van_der1989-1e
Lei,van_der1989-1f
Lei,van_der1989-1g
Li2018
Liu2019-1
Liu2019-2
Liu2019-3
Liu2019-4
Lommen1983-1a
Lommen1983-1b
Lommen1983-1c
0.0
0.5
1.0
event rate and 95% confidence interval

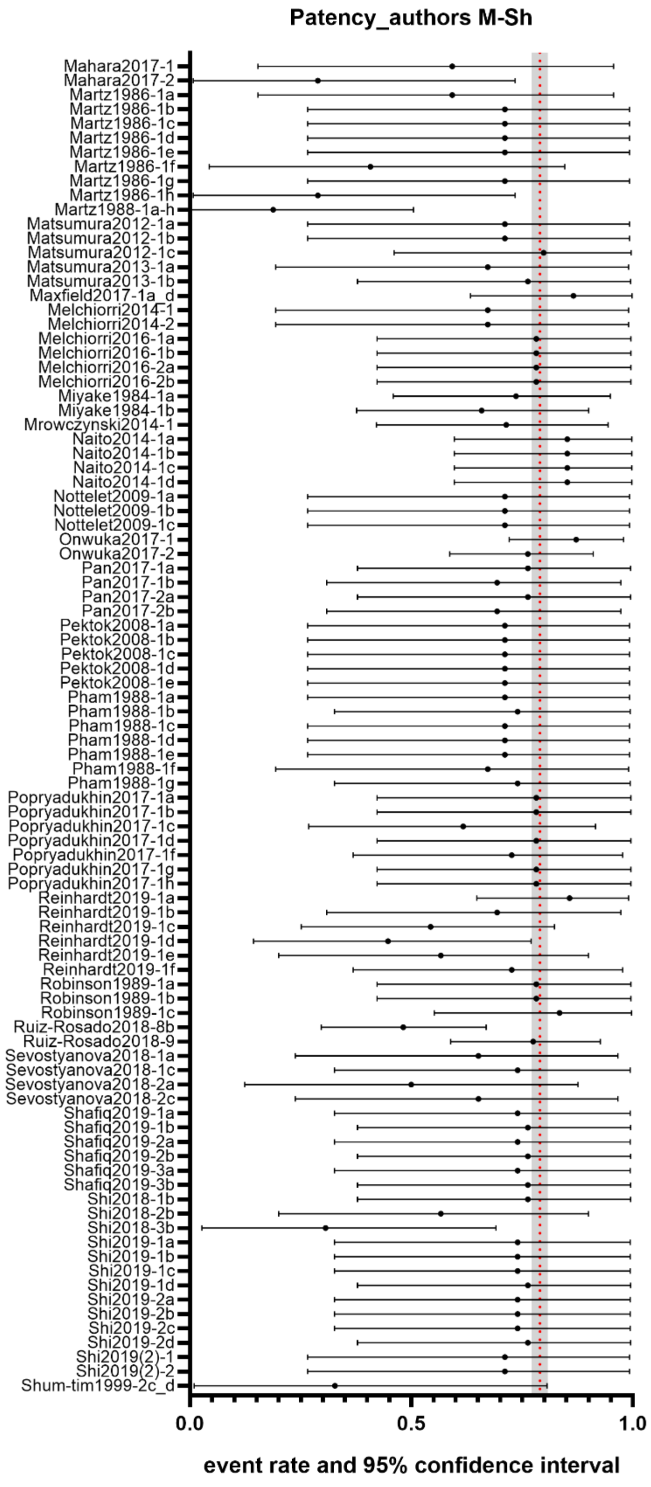
Patency_authors M-Sh
Mahara2017-1
Mahara2017-2
Martz1986-1a
Martz1986-1b
Martz1986-1c
Martz1986-1d
Martz1986-1e
Martz1986-1f
Martz1986-1g
Martz1986-1h
Martz1988-1a-h
Matsumura2012-1a
Matsumura2012-1b
Matsumura2012-1c
Matsumura2013-1a
Matsumura2013-1b
Maxfield2017-1a_d
Melchiorri2014-1
Melchiorri2014-2
Melchiorri2016-1a
Melchiorri2016-1b
Melchiorri2016-2a
Melchiorri2016-2b
Miyake1984-1a
Miyake1984-1b
Mrowczynski2014-1
Naito2014-1a
Naito2014-1b
Naito2014-1c
Naito2014-1d
Nottelet2009-1a
Nottelet2009-1b
Nottelet2009-1c
Onwuka2017-1
Onwuka2017-2
Pan2017-1a
Pan2017-1b
Pan2017-2a
Pan2017-2b
Pektok2008-1a
Pektok2008-1b
Pektok2008-1c
Pektok2008-1d
Pektok2008-1e
Pham1988-1a
Pham1988-1b
Pham1988-1c
Pham1988-1d
Pham1988-1e
Pham1988-1f
Pham1988-1g
Popryadukhin2017-1a
Popryadukhin2017-1b
Popryadukhin2017-1c
Popryadukhin2017-1d
Popryadukhin2017-1f
Popryadukhin2017-1g
Popryadukhin2017-1h
Reinhardt2019-1a
Reinhardt2019-1b
Reinhardt2019-1c
Reinhardt2019-1d
Reinhardt2019-1e
Reinhardt2019-1f
Robinson1989-1a
Robinson1989-1b
Robinson1989-1c
Ruiz-Rosado2018-8b
Ruiz-Rosado2018-9
Sevostyanova2018-1a
Sevostyanova2018-1c
Sevostyanova2018-2a
Sevostyanova2018-2c
Shafiq2019-1a
Shafiq2019-1b
Shafiq2019-2a
Shafiq2019-2b
Shafiq2019-3a
Shafiq2019-3b
Shi2018-1b
Shi2018-2b
Shi2018-3b
Shi2019-1a
Shi2019-1b
Shi2019-1c
Shi2019-1d
Shi2019-2a
Shi2019-2b
Shi2019-2c
Shi2019-2d
Shi2019(2)-1
Shi2019(2)-2
Shum-tim1999-2c_d
0.0
0.5
1.0
event rate and 95% confidence interval

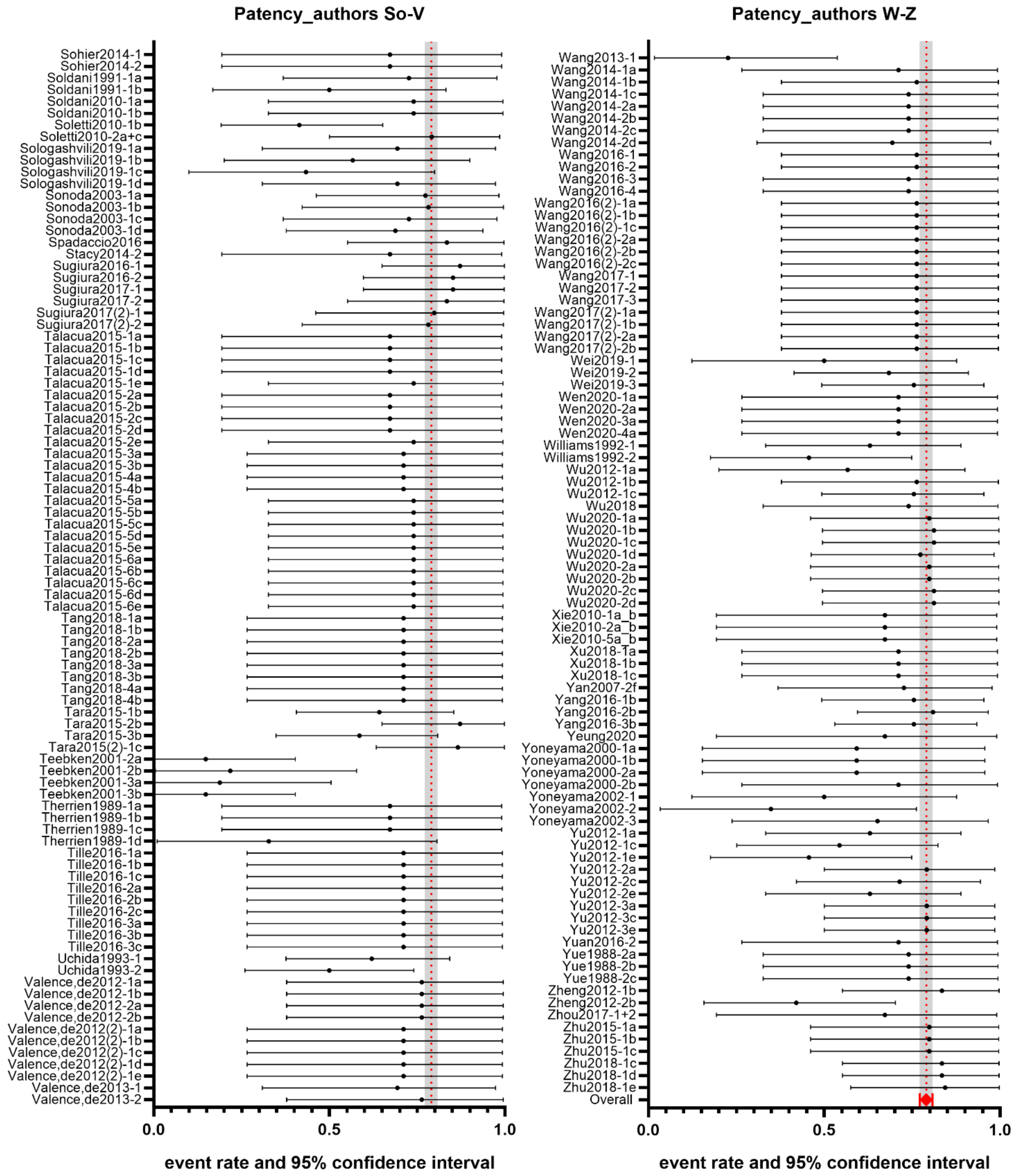

Supplementary Figure 1 Forest plot of overall patency rate of all included experimental groups illustrated as event rate with 95% conficence interval.

a.	Age puberty (average species)	Cut-off value sensitivity analysis young vs adult	Total life expectancy (average species)	Cut-off value sensitivity analysis short vs medium follow up (1.67% of total life expectancy)	Cut-off value sensitivity analysis medium vs long follow up (6.67% of total life expectancy)	References
Mouse	5-7w	7w	2y	14d	60d	[3,4]
Rat	7-11w	11w	3y	21d	90d	[4,5]
Rabbit	3-6m	6m	10y	70d	300d	[4,6,7]
Pig	4-6m	6m	15y	105d	450d	[4,8]
Dog	5-7m	7m	14y	98d	420d	[4,8]
Sheep	n.a.*		12.5y	87d	375d	[4,8]

b.

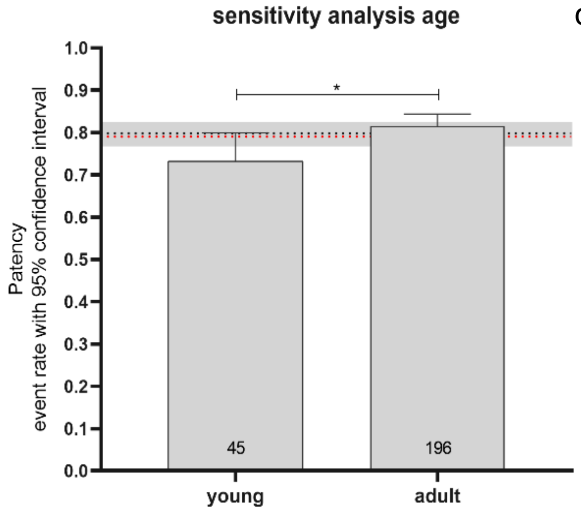

c.

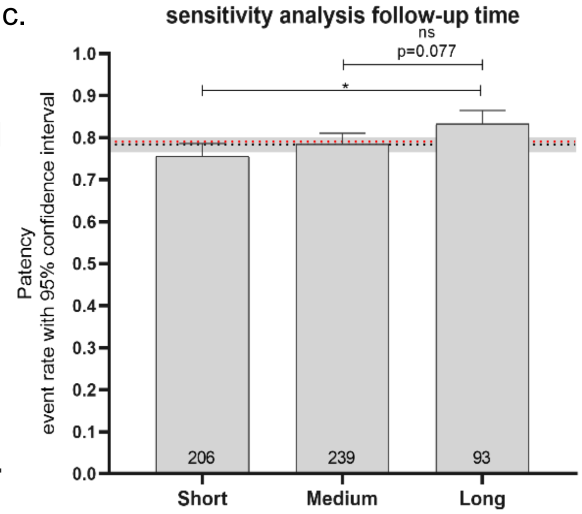

*for sheep studies: method description mentioning "juvenile" or "lamb" was sufficient to make the categorization

Supplementary Figure 2: Sensitivity analysis on subcategorization of age and follow-up time. A) Adjusted cut-off values for subcategorization for sensitivity analysis. B) Sensitivity analysis on age, including period of puberty with young age group, showed no differences compared to previous categorization. C) Sensitivity analysis on follow-up time showed similar trends with previous categorization. Numbers in bar represent number of experimental groups. Black dotted line and grey shades representing grouped ER with 95% CI respectively. Red dotted line: overall ER patency. Significance * $p<0.05$.

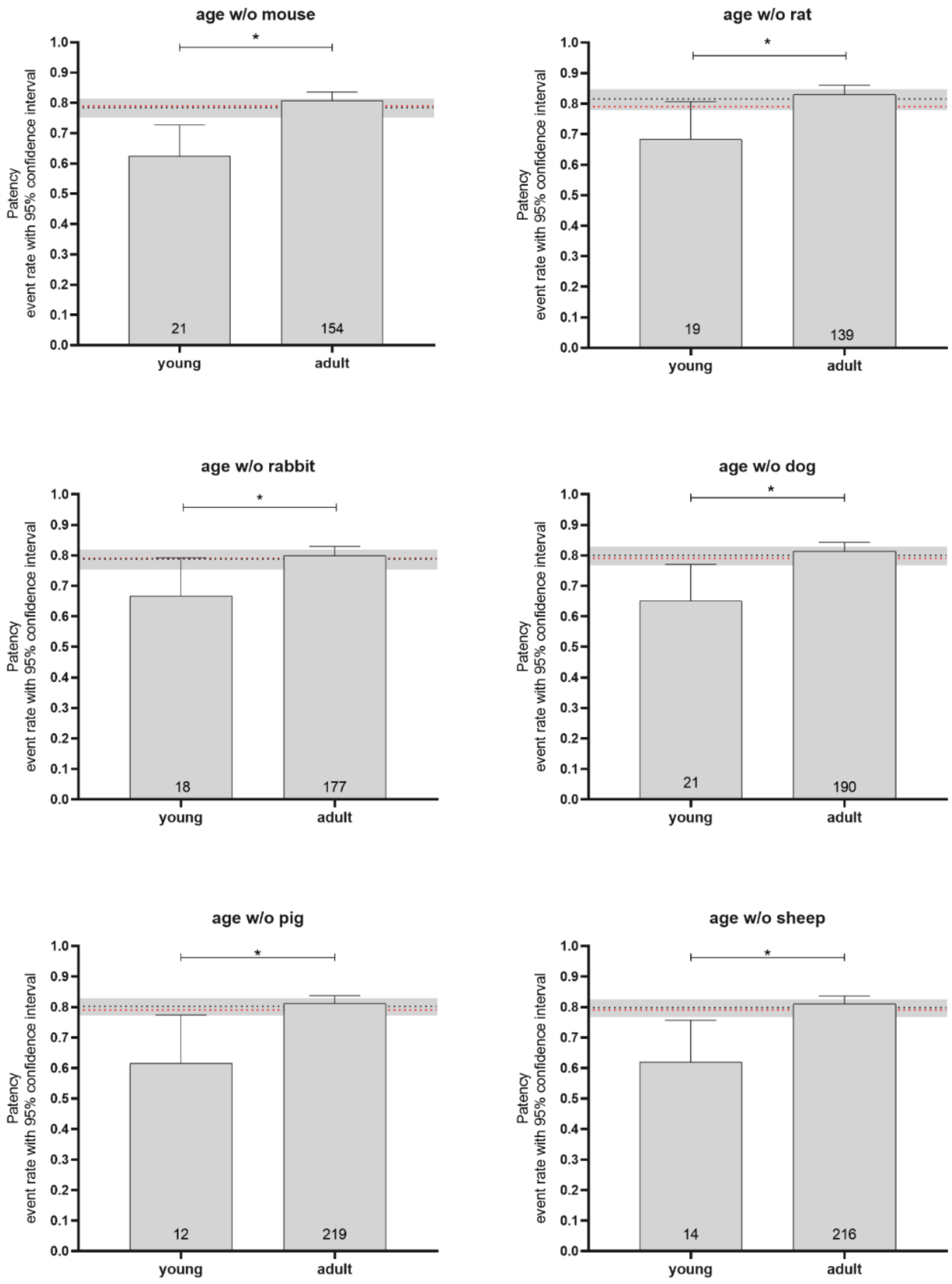

Supplementary Figure 3: Sensitivity analysis of species, showing patency rate per age categorization with exclusion of one species per graph. Numbers in bar represent number of experimental groups. Black dotted line and grey shades representing grouped ER with 95% CI respectively. Red dotted line: overall ER patency. Significance * p<0.05.

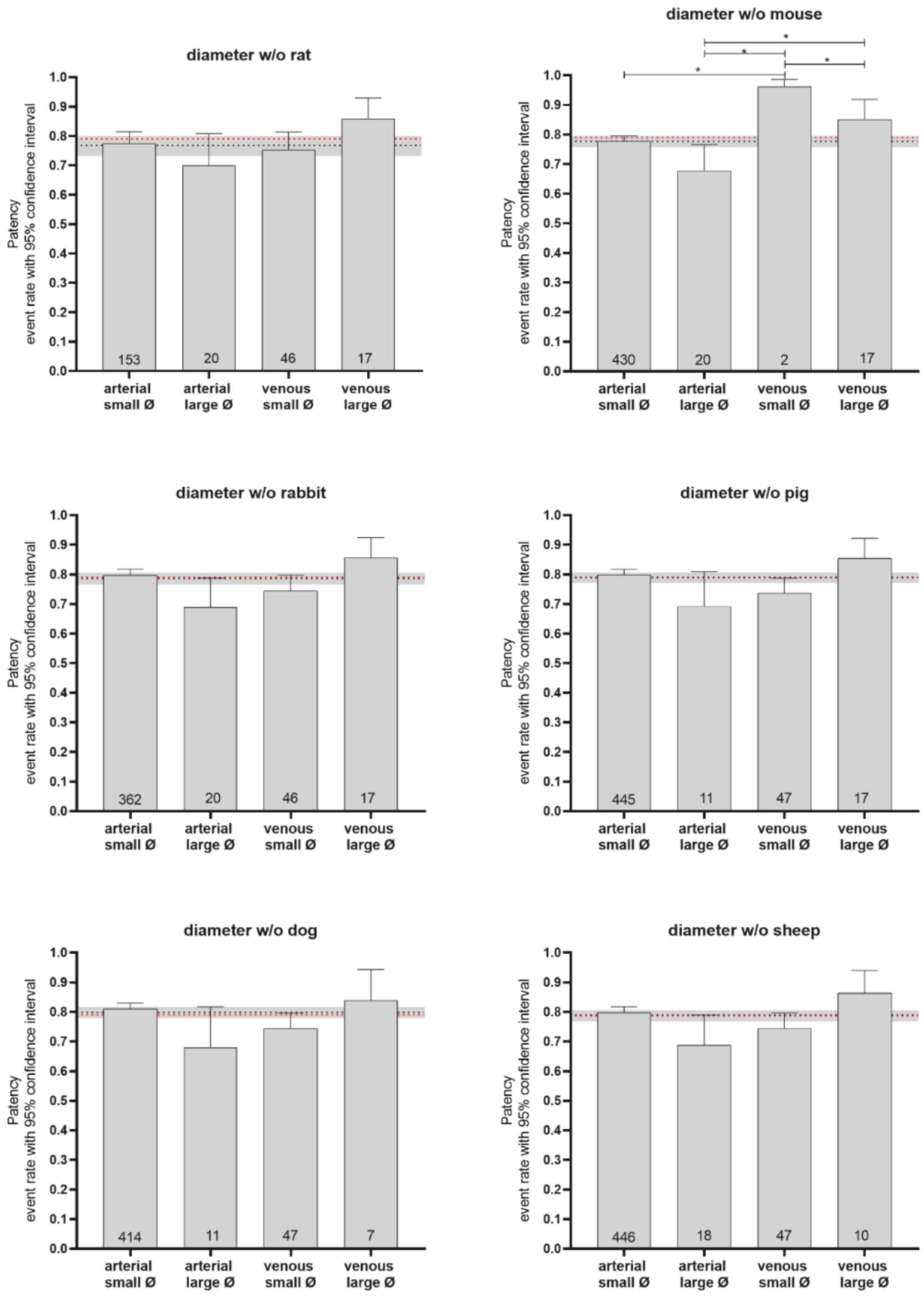

Supplementary Figure 4: Sensitivity analysis of species, showing patency rate per categorization for implant site and graft diameter with exclusion of one species per graph. Numbers in bar represent number of experimental groups. Black dotted line and grey shades representing grouped ER with 95% CI respectively. Red dotted line: overall ER patency. Significance * $p<0.05$

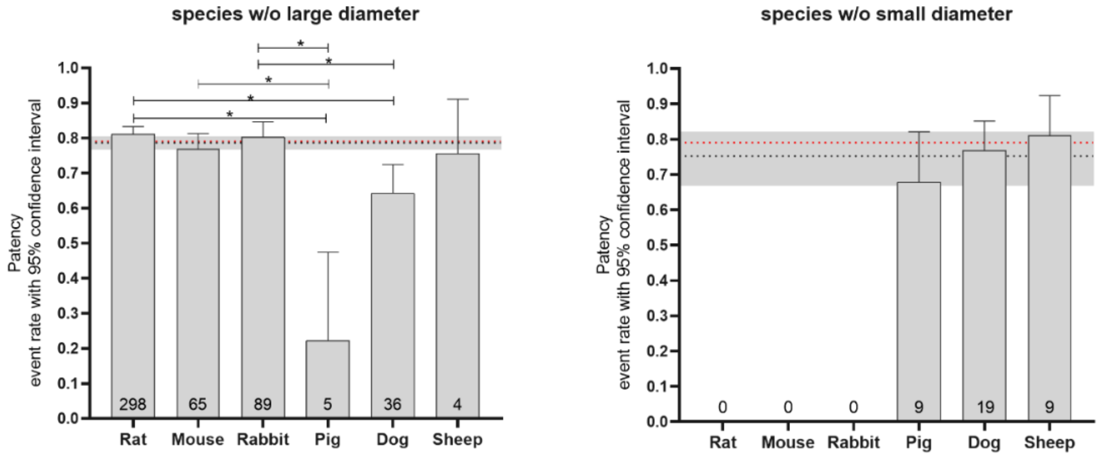

Supplementary Figure 5: Sensitivity analysis of small and large diameter categorization, showing overall patency rate per species for only small diameter grafts (left) or only large diameter grafts (right). Numbers in bar represent number of experimental groups. Black dotted line and grey shades representing grouped ER with 95% CI respectively. Red dotted line: overall ER patency. Significance * $p<0.05$.

Supplementary References: reference list of all 182 included papers.

1. Adler, R. H. & Harrison, J. H. Nylon as a vascular prosthesis in experimental animals with tensile strength studies. Surg Gynecol Obs. 103, 613–617 (1956).
2. Agarwal, R. et al. Degradation and in vivo evaluation of polycaprolactone, poly(ε-caprolactone-co-L-lactide), and poly-L-lactic acid as scaffold sealant polymers for murine tissue-engineered vascular grafts. Regen. Med. 14, 627–637 (2019).
3. Chow, J. P. et al. Mitigation of diabetes-related complications in implanted collagen and elastin scaffolds using matrix-binding polyphenol. Biomaterials 34, 685–695 (2013).
4. van Almen, G. C. et al. Development of Non-Cell Adhesive Vascular Grafts Using Supramolecular Building Blocks. Macromol. Biosci. 16, 350–362 (2016).
5. Antonova, L. V et al. Bioabsorbable Bypass Grafts Biofunctionalised with RGD Have Enhanced Biophysical Properties and Endothelialisation Tested In vivo. Front. Pharmacol. 7, (2016).
6. Antonova, L. V et al. Vascular Endothelial Growth Factor Improves Physico-Mechanical Properties and Enhances Endothelialization of Poly(3-hydroxybutyrate-co-3-hydroxyvalerate)/Poly(ε-caprolactone) Small-Diameter Vascular Grafts In vivo. Front. Pharmacol. 07, (2016)(2).
7. Antonova, L. et al. Conjugation with RGD Peptides and Incorporation of Vascular Endothelial Growth Factor Are Equally Efficient for Biofunctionalization of Tissue-Engineered Vascular Grafts. Int. J. Mol. Sci. 17, 1920 (2016)(3).
8. Antonova, L. et al. Biocompatibility of Small-Diameter Vascular Grafts in Different Modes of RGD Modification. Polymers (Basel). 11, 174 (2019).
9. Antonova, L. V et al. A Brief Report on an Implantation of Small-Caliber Biodegradable Vascular Grafts in a Carotid Artery of the Sheep. Pharmaceuticals 13, 101 (2020).
10. Audell, L., Bowald, S., Busch, C. & Eriksson, I. Polyglactin mesh grafting of the pig aorta. The two-year follow-up in an experimental animal. Acta Chir. Scand. 146, 97–9 (1980).
11. Bartels, H. L. & van der Lei, B. Small-calibre vascular grafting into the rat abdominal aorta with biodegradable prostheses. Lab. Anim. 22, 122–6 (1988).
12. Behr, J. et al. Matching Static and Dynamic Compliance of Small-Diameter Arteries, with Poly(lactide- co - caprolactone) Copolymers: In Vitro and In Vivo Studies. Macromol. Biosci. 20, 1900234 (2020).
13. Bergmeister, H. et al. Biodegradable, thermoplastic polyurethane grafts for small diameter vascular replacements. Acta Biomater. 11, 104–113 (2015).

14. Best, C. et al. Deconstructing the Tissue Engineered Vascular Graft: Evaluating Scaffold Pre-Wetting, Conditioned Media Incubation, and Determining the Optimal Mononuclear Cell Source. ACS Biomater. Sci. Eng. 3, 1972–1979 (2017).

15. Best, C. A. et al. Differential outcomes of venous and arterial tissue engineered vascular grafts highlight the importance of coupling long-term implantation studies with computational modeling. Acta Biomater. 94, 183–194 (2019).

16. Bowald, S., Busch, C. & Eriksson, I. Arterial regeneration following polyglactin 910 suture mesh grafting. Surgery 86, 722–9 (1979).

17. Bowald, S., Busch, C. & Eriksson, I. Absorbable material in vascular prostheses: a new device. Acta Chir. Scand. 146, 391–5 (1980).

18. Brennan, M. P. et al. Tissue-engineered Vascular Grafts Demonstrate Evidence of Growth and Development When Implanted in a Juvenile Animal Model. Ann. Surg. 248, 370–377 (2008).

19. Brothers, T. E., Stanley, J. C., Burkel, W. E. & Graham, L. M. Small-caliber polyurethane and polytetrafluoroethylene grafts: A comparative study in a canine aortoiliac model. J. Biomed. Mater. Res. 24, 761–771 (1990).

20. Brugmans, M., Serrero, A., Cox, M., Svanidze, O. & Schoen, F. J. Morphology and mechanisms of a novel absorbable polymeric conduit in the pulmonary circulation of sheep. Cardiovasc. Pathol. 38, 31–38 (2019).

21. Buscemi, S. et al. Electrospun PHEA-PLA/PCL Scaffold for Vascular Regeneration: A Preliminary in Vivo Evaluation. Transplant. Proc. 49, 716–721 (2017).

22. Chan, A. H. P. et al. Evaluation of synthetic vascular grafts in a mouse carotid grafting model. PLoS One 12, e0174773 (2017).

23. Conconi, M. T. et al. Evaluation of vascular grafts based on polyvinyl alcohol cryogels. Mol. Med. Rep. 10, 1329–1334 (2014).

24. Cui, C., Wen, M., Zhou, F., Zhao, Y. & Yuan, X. Target regulation of both VECs and VSMCs by dual-loading miRNA-126 and miRNA-145 in the bilayered electrospun membrane for small-diameter vascular regeneration. J. Biomed. Mater. Res. Part A 107, 371–382 (2019).

25. Drews, J. D. et al. Spontaneous reversal of stenosis in tissue-engineered vascular grafts. Sci. Transl. Med. 12, eaax6919 (2020).

26. Duan, H.-Y. et al. The in vivo characterization of electrospun heparin-bonded polycaprolactone in small-diameter vascular reconstruction. Vascular 23, 358–365 (2015).

27. Duijvelshoff, R. et al. Host Response and Neo-Tissue Development during Resorption of a Fast Degrading Supramolecular Electrospun Arterial Scaffold. Bioengineering 5, 61 (2018).

28. Eilenberg, M. et al. Long Term Evaluation of Nanofibrous, Bioabsorbable Polycarbonate Urethane Grafts for Small Diameter Vessel Replacement in Rodents. Eur. J. Vasc. Endovasc. Surg. 59, 643–652 (2020).

29. Enayati, M. et al. Biocompatibility Assessment of a New Biodegradable Vascular Graft via In Vitro Co-culture Approaches and In Vivo Model. Ann. Biomed. Eng. 44, 3319–3334 (2016).

30. Fröhlich, S. M. et al. Mass spectrometric imaging of in vivo protein and lipid adsorption on biodegradable vascular replacement systems. Analyst 140, 6089–6099 (2015).

31. Fukunishi, T. et al. Preclinical study of patient-specific cell-free nanofiber tissue-engineered vascular grafts using 3-dimensional printing in a sheep model. J. Thorac. Cardiovasc. Surg. 153, 924–932 (2017).

32. Fukunishi, T. et al. Role of Bone Marrow Mononuclear Cell Seeding for Nanofiber Vascular Grafts. Tissue Eng. Part A 24, 135–144 (2018).

33. Fukunishi, T. et al. Formation of Neoarteries with Optimal Remodeling Using Rapidly Degrading Textile Vascular Grafts. Tissue Eng. Part A 25, 632–641 (2019).

34. Galletti, P. M. et al. Coated bioresorbable mesh as vascular graft material. Trans. Am. Soc. Artif. Intern. Organs 31, 257–63 (1985).

35. Galletti, G., Ussia, G. & Farruggia, F. Arterial replacement with a degradable vascular prosthesis. Life Support Syst. 4, 74–76 (1986).

36. Galletti, P. M., Aebischer, P., Sasken, H. F., Goddard, M. B. & Chiu, T. H. Experience with fully bioresorbable aortic grafts in the dog. Surgery 103, 231–41 (1988).

37. Galletti, G., Gogolewski, S., Ussia, G. & Farruggia, F. Long-term patency of regenerated neoaortic wall following the implant of a fully biodegradable polyurethane prosthesis: experimental lipid diet model in pigs. Ann. Vasc. Surg. 3, 236–243 (1989).

38. Galletti, G. et al. Prevention of platelet aggregation by dietary polyunsaturated fatty acids in the biodegradable polyurethane vascular prosthesis: an experimental model in pigs. Ital. J. Surg. Sci. 19, 121–30 (1989)(2).

39. Gao, Y. et al. Pilot Mouse Study of 1 mm Inner Diameter (ID) Vascular Graft Using Electrospun Poly(ester urea) Nanofibers. Adv. Healthc. Mater. 5, 2427–2436 (2016).

40. Gao, J. et al. The grafts modified by heparinization and catalytic nitric oxide generation used for vascular implantation in rats. Regen. Biomater. 5, 105–114 (2018).

41. Greisler, H. P. Arterial regeneration over absorbable prostheses. Arch. Surg. 117, 1425–1431 (1982).

42. Greisler, H. P., Kim, D. U., Price, J. B. & Voorhees Jr, A. B. Arterial Regenerative Activity After Prosthetic Implantation. Arch. Surg. 120, 315 (1985).

43. Greisler, H. P., Schwarcz, T. H., Ellinger, J. & Kim, D. U. Dacron inhibition of arterial regenerative activities. J. Vasc. Surg. 3, 747–56 (1986).

44. Greisler, H. P. et al. Endothelial cell growth factor attachment to biomaterials. ASAIO Trans. 32, 346–9 (1986)(2).

45. Greisler, H. P. et al. Arterial regeneration over polydioxanone prostheses in the rabbit. Arch. Surg. 122, 715–21 (1987).

46. Greisler, H. P. et al. Derivation of neointima in vascular grafts. Circulation 78, I6-12 (1988).

47. Greisler, H. P. et al. Polyglactin 910/polydioxanone bicomponent totally resorbable vascular prostheses. J. Vasc. Surg. 7, 697–705 (1988)(2).

48. Greisler, H. P. et al. The validity of canine platelet aggregometry in predicting vascular graft patency. J. Cardiovasc. Surg. (Torino). 31, 712–8 (1990).

49. Greisler, H. P. et al. Prostacyclin production by blood-contacting surfaces of endothelialized vascular prostheses. J. Cardiovasc. Surg. (Torino). 31, 640–5 (1990)(2).

50. Greisler, H. P. et al. Kinetics of collagen deposition within bioresorbable and nonresorbable vascular prostheses. ASAIO Trans. 37, M472-5 (1991).

51. Greisler, H. P. et al. Effects of hypercholesterolemia on healing of vascular grafts. J. Investig. Surg. 4, 299–312 (1991)(2).

52. Greisler, H. P. et al. Spatial and temporal changes in compliance following implantation of bioresorbable vascular grafts. J. Biomed. Mater. Res. 26, 1449–1461 (1992).

53. Greisler, H. P. et al. Kinetics of cell proliferation as a function of vascular graft material. J. Biomed. Mater. Res. 27, 955–961 (1993).

54. Hao, D. et al. Rapid endothelialization of small diameter vascular grafts by a bioactive integrin-binding ligand specifically targeting endothelial progenitor cells and endothelial cells. Acta Biomater. 108, 178–193 (2020).

55. Harrison, J. H. Synthetic materials as vascular prostheses - I. A comparative study in small vessels of nylon, dacron, orlon, ivalon sponge and teflon. Am. J. Surg. 95, 3–15 (1958).

56. Harrison, J. H. Synthetic materials as vascular prostheses - II. A comparative study of nylon, dacron, orlon, ivalon sponge and teflon in large blood vessels with tensile strength studies. Am. J. Surg. 95, 16–24 (1958)(2).

57. Hashi, C. K. et al. Antithrombogenic property of bone marrow mesenchymal stem cells in nanofibrous vascular grafts. Proc. Natl. Acad. Sci. 104, 11915–11920 (2007).

58. Hashi, C. K. et al. Antithrombogenic Modification of Small-Diameter Microfibrous Vascular Grafts. Arterioscler. Thromb. Vasc. Biol. 30, 1621–1627 (2010).

59. Haskett, D. G. et al. An exploratory study on the preparation and evaluation of a “same-day” adipose stem cell–based tissue-engineered vascular graft. J. Thorac. Cardiovasc. Surg. 156, 1814-1822.e3 (2018).

60. He, W. et al. Tubular nanofiber scaffolds for tissue engineered small-diameter vascular grafts. J. Biomed. Mater. Res. Part A 90A, 205–216 (2008).

61. Henry, J. J. D. et al. Engineering the mechanical and biological properties of nanofibrous vascular grafts for in situ vascular tissue engineering. Biofabrication 9, 035007 (2017).

62. Hibino, N., Shin’oka, T., Matsumura, G., Ikada, Y. & Kurosawa, H. The tissue-engineered vascular graft using bone marrow without culture. J. Thorac. Cardiovasc. Surg. 129, 1064–1070 (2005).

63. Hibino, N. et al. A critical role for macrophages in neovessel formation and the development of stenosis in tissue-engineered vascular grafts. FASEB J. 25, 4253–4263 (2011).

64. Hibino, N. et al. Evaluation of the use of an induced puripotent stem cell sheet for the construction of tissue-engineered vascular grafts. J. Thorac. Cardiovasc. Surg. 143, 696–703 (2012).

65. Hibino, N. et al. The innate immune system contributes to tissue-engineered vascular graft performance. FASEB J. 29, 2431–2438 (2015).

66. Hibino, N. et al. Novel Association of miR-451 with the Incidence of TEVG Stenosis in a Murine Model. Tissue Eng. Part A 22, 75–82 (2016).

67. Hinrichs, W. L. J., Kuit, J., Feil, H., Wildevuur, C. R. H. & Feijen, J. In vivo fragmentation of microporous Polyurethane- and copolyesterether elastomer-based vascular prostheses. Biomaterials 13, 585–593 (1992).

68. Hong, Y. et al. A small diameter, fibrous vascular conduit generated from a poly(ester urethane)urea and phospholipid polymer blend. Biomaterials 30, 2457–2467 (2009).

69. Horakova, J. et al. Electrospun vascular grafts fabricated from poly(L-lactide-co-ϵ-caprolactone) used as a bypass for the rabbit carotid artery. Biomed. Mater. 13, (2018).

70. Huang, R. et al. Triple-Layer Vascular Grafts Fabricated by Combined E-Jet 3D Printing and Electrospinning. Ann. Biomed. Eng. 46, 1254–1266 (2018).

71. Innocente, F. et al. Paclitaxel-Eluting Biodegradable Synthetic Vascular Prostheses: A Step Towards Reduction of Neointima Formation? Circulation 120, S37–S45 (2009).

72. Isayama, N., Matsumura, G., Sato, H., Matsuda, S. & Yamazaki, K. Histological maturation of vascular smooth muscle cells in in situ tissue-engineered vasculature. Biomaterials 35, 3589–3595 (2014).

73. Janairo, R. R. R. et al. Heparin-Modified Small-Diameter Nanofibrous Vascular Grafts. IEEE Trans. Nanobioscience 11, 22–27 (2012).

74. Jang, E. H., Kim, J.-H., Lee, J. H., Kim, D.-H. & Youn, Y.-N. Enhanced Biocompatibility of Multi-Layered, 3D Bio-Printed Artificial Vessels Composed of Autologous Mesenchymal Stem Cells. Polymers (Basel). 12, (2020).

75. Jirofti, N., Mohebbi-Kalhori, D., Samimi, A., Hadjizadeh, A. & Kazemzadeh, G. H. Small-diameter vascular graft using co-electrospun composite PCL/PU nanofibers. Biomed. Mater. 13, 055014 (2018).

76. Kang, T.-Y. et al. In vivo endothelization of tubular vascular grafts through in situ recruitment of endothelial and endothelial progenitor cells by RGD-fused mussel adhesive proteins. Biofabrication 7, 015007 (2015).

77. Khosravi, R. et al. Biomechanical diversity despite mechanobiological stability in tissue engineered vascular grafts two years post-implantation. Tissue Eng. - Part A 21, 1529–1538 (2015).

78. Khosravi, R. et al. Long-Term Functional Efficacy of a Novel Electrospun Poly(Glycerol Sebacate)-Based Arterial Graft in Mice. Ann. Biomed. Eng. 44, 2402–2416 (2016).

79. Kurobe, H. et al. Development of small diameter nanofiber tissue engineered arterial grafts. PLoS One 10 (4) (no, (2015).

80. Kuwabara, F. et al. Novel small-caliber vascular grafts with trimeric peptide for acceleration of endothelialization. Ann. Thorac. Surg. 93, 156–163 (2012).

81. Kuwabara, F. et al. Long-term results of tissue-engineered small-caliber vascular grafts in a rat carotid arterial replacement model. J. Artif. Organs 15, 399–405 (2012)(2).

82. Lauritzen, C. Experimental Studies on Absorbable Vascular Grafts for Microsurgery. Scand. J. Plast. Reconstr. Surg. 17, 133–135 (1983).

83. Lee, Y. et al. TGF-β receptor 1 inhibition prevents stenosis of tissue-engineered vascular grafts by reducing host mononuclear phagocyte activation. FASEB J. 30, 2627–2636 (2016).

84. Lee, Y. U. et al. Rational design of an improved tissue-engineered vascular graft: Determining the optimal cell dose and incubation time. Regen. Med. 11, 159–167 (2016)(2).

85. Lee, K.-W. et al. A biodegradable synthetic graft for small arteries matches the performance of autologous vein in rat carotid arteries. Biomaterials 181, 67–80 (2018).

86. van der Lei, B. et al. Regeneration of the arterial wall in microporous, compliant, biodegradable vascular grafts after implantation into the rat abdominal aorta. Cell Tissue Res. 242, 569–578 (1985).

87. van der Lei, B. et al. Arterial wall regeneration in small-caliber vascular grafts in rats. Neoendothelial healing and prostacyclin production. J. Thorac. Cardiovasc. Surg. 90, 378–86 (1985)(2).

88. van der Lei, B., Bartels, H. L., Nieuwenhuis, P. & Wildevuur, C. R. Microporous, complaint, biodegradable vascular grafts for the regeneration of the arterial wall in rat abdominal aorta. Surgery 98, 955–63 (1985)(3).

89. van der Lei, B., Wildevuur, C. R. & Nieuwenhuis, P. Compliance and biodegradation of vascular grafts stimulate the regeneration of elastic laminae in neoarterial tissue: an experimental study in rats. Surgery 99, 45–52 (1986).

90. van der Lei, B. et al. Sequential studies of arterial wall regeneration in microporous, compliant, biodegradable small-caliber vascular grafts in rats. J. Thorac. Cardiovasc. Surg. 93, 695–707 (1987).

91. van der Lei, B., Nieuwenhuis, P., Molenaar, I. & Wildevuur, C. R. Long-term biologic fate of neoarteries regenerated in microporous, compliant, biodegradable, small-caliber vascular grafts in rats. Surgery 101, 459–67 (1987)(2).

92. van der Lei, B., Robinson, P. H., Bartels, H. L. & Wildevuur, C. R. Microarterial grafting into the carotid artery of the rabbit: some considerations concerning species-dependent thrombogenicity. Br. J. Plast. Surg. 42, 59–64 (1989).

93. Li, W. et al. Long-term evaluation of vascular grafts with circumferentially aligned microfibers in a rat abdominal aorta replacement model. J. Biomed. Mater. Res. Part B Appl. Biomater. 106, 2596–2604 (2018).

94. Liu, J. et al. The surrounding tissue contributes to smooth muscle cells' regeneration and vascularization of small diameter vascular grafts. Biomater. Sci. 7, 914–925 (2019).

95. Lommen, E., Gogolewski, S., Pennings, A. J., Wildevuur, C. R. & Nieuwenhuis, P. Development of a neo-artery induced by a biodegradable polymeric vascular prosthesis. Trans. Am. Soc. Artif. Intern. Organs 29, 255–9 (1983).

96. Mahara, A., Kiick, K. L. & Yamaoka, T. In vivo guided vascular regeneration with a non-porous elastin-like polypeptide hydrogel tubular scaffold. J. Biomed. Mater. Res. - Part A 105, 1746–1755 (2017).

97. Martz, H., Paynter, R., Forest, J.-C., Downs, A. & Guidoin, R. Microporous hydrophilic polyurethane vascular grafts as substitutes in the abdominal aorta of dogs. Biomaterials 8, 3–11 (1986).

98. Martz, H. et al. Hydrophilic microporous polyurethane versus expanded PTFE grafts as substitutes in the carotid arteries of dogs. A limited study. J. Biomed. Mater. Res. 22, 63–69 (1988).

99. Matsumura, G. et al. Evaluation of tissue-engineered vascular autografts. Tissue Eng. 12, 3075–3083 (2006).

100. Matsumura, G. et al. Long-term results of cell-free biodegradable scaffolds for in situ tissue-engineering vasculature: in a canine inferior vena cava model. PLoS One 7, e35760 (2012).

101. Matsumura, G. et al. Long-term results of cell-free biodegradable scaffolds for in situ tissue engineering of pulmonary artery in a canine model. Biomaterials 34, 6422–6428 (2013).

102. Maxfield, M. W. et al. Novel application and serial evaluation of tissue-engineered portal vein grafts in a murine model. Regen. Med. 12, 929–938 (2017).

103. Melchiorri, A. J., Hibino, N., Brandes, Z. R., Jonas, R. A. & Fisher, J. P. Development and assessment of a biodegradable solvent cast polyester fabric small-diameter vascular graft. J. Biomed. Mater. Res. Part A 102, 1972–1981 (2014).

104. Melchiorri, A. J. et al. Contrasting biofunctionalization strategies for the enhanced endothelialization of biodegradable vascular grafts. Biomacromolecules 16, 437–446 (2015).

105. Melchiorri, A. J. et al. 3D-Printed Biodegradable Polymeric Vascular Grafts. Adv. Healthc. Mater. 5, 319–325 (2016).

106. Miyake, H. et al. New small-caliber antithrombotic vascular prosthesis: Experimental study. Microsurgery 5, 144–150 (1984).

107. Mrówczyński, W. et al. Porcine carotid artery replacement with biodegradable electrospun poly-e-caprolactone vascular prosthesis. J. Vasc. Surg. 59, 210–219 (2014).

108. Mun, C. H. et al. Elastic, double-layered poly (l-lactide-co-ϵ-caprolactone) scaffold for long-term vascular reconstruction. J. Bioact. Compat. Polym. 28, 233–246 (2013).

109. Muylaert, D. E. P. et al. Early in-situ cellularization of a supramolecular vascular graft is modified by synthetic stromal cell-derived factor-1α derived peptides. Biomaterials 76, 187–195 (2016).

110. Naito, Y. et al. Beyond burst pressure: Initial evaluation of the natural history of the biaxial mechanical properties of tissue-engineered vascular grafts in the venous circulation using a murine model. Tissue Eng. - Part A 20, 346–355 (2014).

111. Nottelet, B. et al. Factorial design optimization and in vivo feasibility of poly(ε-caprolactone)-micro- and nanofiber-based small diameter vascular grafts. J. Biomed. Mater. Res. Part A 89A, 865–875 (2009).

112. Onwuka, E. et al. The role of myeloid cell-derived PDGF-B in neotissue formation in a tissue-engineered vascular graft. Regen. Med. 12, 249–261 (2017).

113. Pan, Y. et al. Small-diameter hybrid vascular grafts composed of polycaprolactone and polydioxanone fibers. Sci. Rep. 7, 3615 (2017).

114. Pektok, E. et al. Degradation and Healing Characteristics of Small-Diameter Poly(ε-Caprolactone) Vascular Grafts in the Rat Systemic Arterial Circulation. Circulation 118, 2563–2570 (2008).

115. Pepper, V. K. et al. Intravascular Ultrasound Characterization of a Tissue-Engineered Vascular Graft in an Ovine Model. J. Cardiovasc. Transl. Res. 10, 128–138 (2017).

116. Pham SJ, D. S. et al. Compliance changes in bioresorbable vascular prostheses after implantation. Surg. Forum 29, 330–332 (1988).

117. Popryadukhin, P. V et al. Tissue-Engineered Vascular Graft of Small Diameter Based on Electrospun Polylactide Microfibers. Int. J. Biomater. 2017 (no p, (2017).

118. Reinhardt, J. W. et al. Early natural history of neotissue formation in tissue-engineered vascular grafts in a murine model. Regen. Med. 14, 389–408 (2019).

119. Robinson, P. H., Van Der Lei, B., Knol, K. E. & Pennings, A. J. Patency and long-term biological fate of a two-ply biodegradable microaterial prosthesis in the rat. Br. J. Plast. Surg. 42, 544–549 (1989).

120. Roh, J. D. et al. Small-diameter biodegradable scaffolds for functional vascular tissue engineering in the mouse model. Biomaterials 29, 1454–1463 (2008).

121. Ruiz-Rosado, J. de D. et al. Angiotensin II receptor I blockade prevents stenosis of tissue engineered vascular grafts. FASEB J. Off. Publ. Fed. Am. Soc. Exp. Biol. fj201800458 (2018) doi:10.1096/fj.201800458.

122. Sevostyanova, V. et al. Use of polycaprolactone grafts for small-diameter blood vessels. Angiol. Vasc. Surg. 21, 44–53 (2015).

123. Sevostyanova, V. V et al. Endothelialization of Polycaprolactone Vascular Graft under the Action of Locally Applied Vascular Endothelial Growth Factor. Bull. Exp. Biol. Med. 165, 264–268 (2018).

124. Shafiq, M. et al. In situ blood vessel regeneration using neuropeptide substance P-conjugated small-diameter vascular grafts. J. Biomed. Mater. Res. Part B Appl. Biomater. 107, 1669–1683 (2019).

125. Shi, J., Zhang, J., Yin, M., Wang, Q. & Du, J. Accurate and continuous ultrasonography evaluation of small diameter vascular prostheses in vivo. Exp. Ther. Med. 15, 3899–3907 (2018).

126. Shi, J. et al. Regulation of the inflammatory response by vascular grafts modified with Aspirin-Triggered Resolvin D1 promotes blood vessel regeneration. Acta Biomater. 97, 360–373 (2019).

127. Shi, J. et al. Rapid endothelialization and controlled smooth muscle regeneration by electrospun heparin-loaded polycaprolactone/gelatin hybrid vascular grafts. J. Biomed. Mater. Res. Part B Appl. Biomater. 1–10 (2018)(2).

128. Shinoka, T. et al. Creation of viable pulmonary artery autografts through tissue engineering. J. Thorac. Cardiovasc. Surg. 115, 536–546 (1998).

129. Shum-Tim, D. et al. Tissue engineering of autologous aorta using a new biodegradable polymer. Ann. Thorac. Surg. 68, 2298–2304 (1999).

130. Sohier, J., Corre, P., Perret, C., Pilet, P. & Weiss, P. Novel and Simple Alternative to Create Nanofibrillar Matrices of Interest for Tissue Engineering. Tissue Eng. Part C Methods 20, 285–296 (2014).

131. Soldani, G. & Mercogliano, R. Bioartificial polymeric materials obtained from blends of synthetic polymers with fibrin and collagen. Int. J. Artif. Organs 14, 295–303 (1991).

132. Soldani, G. et al. Long term performance of small-diameter vascular grafts made of a poly(ether)urethane–polydimethylsiloxane semi-interpenetrating polymeric network. Biomaterials 31, 2592–2605 (2010).

133. Soletti, L. et al. In vivo performance of a phospholipid-coated bioerodable elastomeric graft for small-diameter vascular applications. J. Biomed. Mater. Res. Part A 96A, 436–448 (2010).

134. Sologashvili, T. et al. European Journal of Pharmaceutics and Biopharmaceutics E ff ect of implantation site on outcome of tissue-engineered vascular grafts. Eur. J. Pharm. Biopharm. 139, 272–278 (2019).

135. Sonoda, H., Takamizawa, K., Nakayama, Y., Yasui, H. & Matsuda, T. Coaxial double-tubular compliant arterial graft prosthesis: Time-dependent morphogenesis and compliance changes after implantation. J. Biomed. Mater. Res. 65A, 170–181 (2003).

136. Spadaccio, C. et al. Preliminary in vivo evaluation of a hybrid armored vascular graft combining electrospinning and additive manufacturing techniques. Drug Target Insights 10, 1–7 (2016).

137. Stacy, M. R. et al. Targeted imaging of matrix metalloproteinase activity in the evaluation of remodeling tissue-engineered vascular grafts implanted in a growing lamb model. J. Thorac. Cardiovasc. Surg. 148, 2227–2233 (2014).

138. Stacy, M. R. et al. Magnetic Resonance Imaging of Shear Stress and Wall Thickness in Tissue-Engineered Vascular Grafts. Tissue Eng. Part C Methods 24, 465–473 (2018).

139. Sugiura, T. et al. Novel Bioresorbable Vascular Graft With Sponge-Type Scaffold as a Small-Diameter Arterial Graft. Ann. Thorac. Surg. 102, 720–727 (2016).

140. Sugiura, T. et al. Fast-degrading bioresorbable arterial vascular graft with high cellular infiltration inhibits calcification of the graft. J. Vasc. Surg. 66, 243–250 (2017).

141. Sugiura, T. et al. Tropoelastin inhibits intimal hyperplasia of mouse bioresorbable arterial vascular grafts. Acta Biomater. 52, 74–80 (2017)(2).

142. Talacua, H. et al. In Situ Tissue Engineering of Functional Small-Diameter Blood Vessels by Host Circulating Cells Only. Tissue Eng. Part A 21, 2583–2594 (2015).

143. Tan, R. P. et al. Bioactive Materials Facilitating Targeted Local Modulation of Inflammation. JACC Basic to Transl. Sci. 4, 56–71 (2019).

144. Tang, D. et al. Materials Science & Engineering C Regulation of macrophage polarization and promotion of endothelialization by NO generating and PEG-YIGSR modi fi ed vascular graft. Mater. Sci. Eng. C 84, 1–11 (2018).

145. Tara, S. et al. Well-organized neointima of large-pore poly(l-lactic acid) vascular graft coated with poly(l-lactic-co-ε-caprolactone) prevents calcific deposition compared to small-pore electrospun poly(l-lactic acid) graft in a mouse aortic implantation model. Atherosclerosis 237, 684–691 (2014).

146. Tara, S. et al. Cilostazol, Not Aspirin, Prevents Stenosis of Bioresorbable Vascular Grafts in a Venous Model. Arterioscler. Thromb. Vasc. Biol. 35, 2003–2010 (2015).

147. Tara, S. et al. Evaluation of remodeling process in small-diameter cell-free tissue-engineered arterial graft. J. Vasc. Surg. 62, 734–743 (2015).

148. Teebken, O. E., Pichlmaier, A. M. & Haverich, A. Cell seeded decellularised allogeneic matrix grafts and biodegradable polydioxanone-prostheses compared with arterial autografts in a porcine model. Eur. J. Vasc. Endovasc. Surg. 22, 139–145 (2001).

149. Therrien, M., Guidoin, R. G., Adnot, A. & Paynter, R. Hydrophobic and fibrillar microporous polyetherurethane urea prosthesis: an ESCA study on the internal and external surfaces of explanted grafts. Biomaterials 10, 517–520 (1989).

150. Tille, J.-C. et al. Histologic Assessment of Drug-Eluting Grafts Related to Implantation Site. J. Dev. Biol. 4, (2016).

151. Uchida, N. et al. Compliance effects on small diameter polyurethane graft patency. J. Biomed. Mater. Res. 27, 1269–79 (1993).

152. Valence, S. De et al. Acta Biomaterialia Advantages of bilayered vascular grafts for surgical applicability and tissue regeneration. Acta Biomater. 8, 3914–3920 (2012).

153. de Valence, S. et al. Long term performance of polycaprolactone vascular grafts in a rat abdominal aorta replacement model. Biomaterials 33, 38–47 (2012)(2).

154. Valence, S. De et al. Plasma treatment for improving cell biocompatibility of a biodegradable polymer scaffold for vascular graft applications. Eur. J. Pharm. Biopharm. 85, 78–86 (2013).

155. Wang, S. et al. Fabrication of small-diameter vascular scaffolds by heparin-bonded P(LLA-CL) composite nanofibers to improve graft patency. Int. J. Nanomedicine 8, 2131 (2013).

156. Wang, Z. et al. The effect of thick fibers and large pores of electrospun poly(ε-caprolactone) vascular grafts on macrophage polarization and arterial regeneration. Biomaterials 35, 5700–10 (2014).

157. Wang, Y. et al. Rapid in situ endothelialization of a small diameter vascular graft with catalytic nitric oxide generation and promoted endothelial cell adhesion. J. Mater. Chem. B 3, 9212–9222 (2015).

158. Wang, Z. et al. Differences in the performance of PCL-based vascular grafts as abdominal aorta substitutes in healthy and diabetic rats. Biomater. Sci. 4, 1485–1492 (2016).

159. Wang, K. et al. Three-Layered PCL Grafts Promoted Vascular Regeneration in a Rabbit Carotid Artery Model. Macromol. Biosci. 16, 608–918 (2016)(2).

160. Wang, K. et al. Functional Modification of Electrospun Poly(ε-caprolactone) Vascular Grafts with the Fusion Protein VEGF–HGFI Enhanced Vascular Regeneration. ACS Appl. Mater. Interfaces 9, 11415–11427 (2017).

161. Wang, Z. et al. Effect of Resveratrol on Modulation of Endothelial Cells and Macrophages for Rapid Vascular Regeneration from Electrospun Poly(ε-caprolactone) Scaffolds. ACS Appl. Mater. Interfaces 9, 19541–19551 (2017)(2).

162. Wei, Y. et al. MSC-derived sEVs enhance patency and inhibit calcification of synthetic vascular grafts by immunomodulation in a rat model of hyperlipidemia. Biomaterials 204, 13–24 (2019).

163. Wen, M. et al. Local Delivery of Dual MicroRNAs in Trilayered Electrospun Grafts for Vascular Regeneration. ACS Appl. Mater. Interfaces 12, 6863–6875 (2020).

164. Williams, S. K. et al. Formation of a multilayer cellular lining on a polyurethane vascular graft following endothelial cell sodding. J. Biomed. Mater. Res. 26, 103–117 (1992).

165. Wise, S. G. et al. A multilayered synthetic human elastin/polycaprolactone hybrid vascular graft with tailored mechanical properties. Acta Biomater. 7, 295–303 (2011).

166. Wu, W., Allen, R. A. & Wang, Y. Fast-degrading elastomer enables rapid remodeling of a cell-free synthetic graft into a neoartery. Nat. Med. 18, 1148–53 (2012).

167. Wu, Y. et al. The regeneration of macro-porous electrospun poly(ε-caprolactone) vascular graft during long-term in situ implantation. J. Biomed. Mater. Res. - Part B Appl. Biomater. 106, 1618–1627 (2018).

168. Wu, P. et al. Construction of vascular graft with circumferentially oriented microchannels for improving artery regeneration. Biomaterials 242, 119922 (2020).

169. Xie, X. et al. Five Types of Polyurethane Vascular Grafts in Dogs: The Importance of Structural Design and Material Selection. J. Biomater. Sci. Polym. Ed. 21, 1239–1264 (2010).

170. Xu, Z. et al. Vascular Remodeling Process of Heparin-Conjugated Poly(ε-Caprolactone) Scaffold in a Rat Abdominal Aorta Replacement Model. J. Vasc. Res. 55, 338–349 (2018).

171. Yan, Y., Hong Wang, X., Yin, D. & Zhang, R. A New Polyurethane/Heparin Vascular Graft for Small-Caliber Vein Repair. J. Bioact. Compat. Polym. 22, 323–341 (2007).

172. Yang, X., Wei, J., Lei, D., Liu, Y. & Wu, W. Appropriate density of PCL nano-fiber sheath promoted muscular remodeling of PGS/PCL grafts in arterial circulation. Biomaterials 88, 34–47 (2016).

173. Yeung, E. et al. In vivo implantation of 3-dimensional printed customized branched tissue engineered vascular graft in a porcine model. J. Thorac. Cardiovasc. Surg. 159, 1971-1981.e1 (2020).

174. Yoneyama, T., Ito, M., Sugihara, K., Ishihara, K. & Nakabayashi, N. Small Diameter Vascular Prosthesis with a Nonthrombogenic Phospholipid Polymer Surface: Preliminary Study of a New Concept for Functioning in the Absence of Pseudo- or Neointima Formation. Artif. Organs 24, 23–28 (2000).

175. Yoneyama, T., Sugihara, K., Ishihara, K., Iwasaki, Y. & Nakabayashi, N. The vascular prosthesis without pseudointima prepared by antithrombogenic phospholipid polymer. Biomaterials 23, 1455–1459 (2002).

176. Yu, J. et al. The effect of stromal cell-derived factor-1alpha/heparin coating of biodegradable vascular grafts on the recruitment of both endothelial and smooth muscle progenitor cells for accelerated regeneration. Biomaterials 33, 8062–8074 (2012).

177. Yuan, H. et al. Highly aligned core–shell structured nanofibers for promoting phenotypic expression of vSMCs for vascular regeneration. Nanoscale 8, 16307–16322 (2016).

178. Yue, X. et al. Smooth muscle cell seeding in biodegradable grafts in rats: A new method to enhance the process of arterial wall regeneration. Surgery 103, 206–212 (1988).

179. Zheng, W. et al. Endothelialization and patency of RGD-functionalized vascular grafts in a rabbit carotid artery model. Biomaterials 33, 2880–2891 (2012).

180. Zhou, P., Zhou, F., Liu, B., Zhao, Y. & Yuan, X. Functional electrospun fibrous scaffolds with dextran- g -poly(l -lysine)-VAPG/microRNA-145 to specially modulate vascular SMCs. J. Mater. Chem. B 5, 9312–9325 (2017).

181. Zhu, M. et al. Circumferentially aligned fibers guided functional neoartery regeneration in vivo. Biomaterials 61, 85–94 (2015).

182. Zhu, M. et al. Biodegradable and elastomeric vascular grafts enable vascular remodeling. Biomaterials 183, 306–318 (2018).

References to the Supplemental Information

1. de Vries RBM, Hooijmans CR, Tillema A, Leenaars M, Ritskes-Hoitinga M. A search filter for increasing the retrieval of animal studies in Embase. *Lab Anim*. 2011;45(4):268-270.
2. Hooijmans CR, Tillema A, Leenaars M, Ritskes-Hoitinga M. Enhancing search efficiency by means of a search filter for finding all studies on animal experimentation in PubMed. *Lab Anim*. 2010;44(3):170-175.
3. Dutta S, Sengupta P. Men and mice: Relating their ages. *Life Sci*. 2016;152(May):244-248.
4. Kim NN, Parker RM, Weinbauer GF, Remick AK, Steinbach T. Points to Consider in Designing and Conducting Juvenile Toxicology Studies. *Int J Toxicol*. 2017;36(4):325-339.
5. Sengupta P. The laboratory rat: Relating its age with human's. *Int J Prev Med*. 2013;4(6):624-630.
6. Dutta S, Sengupta P. Rabbits and men: Relating their ages. *J Basic Clin Physiol Pharmacol*. 2018;29(5):427-435.
7. Sengupta P, Dutta S. Mapping the Age of Laboratory Rabbit Strains to Human. *Int J Prev Med*. 2020;11(December):194.
8. Cummings GD. *Guide for the Care and Use of Laboratory Animals*. Vol 38. Washington, D.C.: National Academies Press; 2011.
